# LKB1 promotes lung adenocarcinoma cell senescence by suppressing the O-GlcNAc glycosylation-mediated activation of telomerase

**DOI:** 10.3389/fphar.2026.1952846

**Published:** 2026-09-28

**Authors:** Liting Gu, Mingdi Liu, Yunkuo Li, Honglan Zhou, Yishu Wang, Zhi-Xiang Xu

**Affiliations:** 1 Key Laboratory of Pathobiology, Ministry of Education, Jilin University, Jilin, China; 2 Department of Urology, The First Hospital of Jilin University, Jilin, China

**Keywords:** cellular senescence, LKB1, lung adenocarcinoma, O-GlcNAc glycosylation, telomerase

## Abstract

**Background:**

Telomeres are crucial for maintaining genomic integrity and stability, making them promising anticancer therapy targets. O-GlcNAc glycosylation is unique and involved in most cellular functions. In most cancers, there is a positive correlation between the degree of tumor malignancy and O-GlcNAc glycosylation levels. However, the relationship between O-GlcNAc glycosylation and telomerase in lung adenocarcinoma remains unclear. We thus investigated the O-GlcNAc-mediated regulation of telomerase activity.

**Methods:**

Protein and mRNA expression levels were detected *via* western blotting and RT-qPCR, respectively. Telomerase activity, telomere length, and telomere-induced injury foci were detected using telomeric repeat amplification protocol, telomere restriction fragment, and Q-FISH assays, respectively. Cell senescence and proliferation were detected using SA-β-gal staining and EdU assays. IHC and H&E staining were used to detect p53, PCNA, and BCL-2 protein levels in the transplanted tumor tissues and their effects on organs in transplanted tumor-bearing mice.

**Results:**

Our findings revealed that LKB1 reduces the overall activity of O-GlcNAcylation and telomerase, resulting in the shortening of telomeres, increased senescence and apoptosis, and decreased tumor cell growth. Insights into the underlying mechanisms have shown that LKB1–AMPK influence the hexosamine biosynthesis pathway (HBP), potentially *via* the phosphorylation of GFAT1. *In vivo* studies revealed that using both O-GlcNAc and telomerase inhibitors together led to the senescence of tumor cells and hindered their growth, suggesting a viable treatment method for clinical lung adenocarcinoma.

**Discussion:**

O-GlcNAc glycosylation promotes telomerase activity and inhibits tumor cell senescence, thereby maintaining telomere lengths in lung adenocarcinoma tumor cells. LKB1 inhibits telomerase activity *via* the HBP, which may depend on GFAT1 phosphorylation by LKB1. Telomerase inhibitors coupled with O-GlcNAc inhibitors significantly inhibit tumor growth in mice, suggesting clinical lung adenocarcinoma treatment strategies.

## 1 Introduction

Glycosylation constitutes a pivotal post-translational modification that critically modulates protein structure, stability, and functional diversity within the intracellular environment (He et al., 2024). Glucose is transported into the cell through glucose transporters, but only a small percentage (2%–5%) of glucose enters the hexosamine biosynthesis pathway (HBP) to produce ATP. The HBP is a significant metabolic pathway that regulates nucleotide sugar synthesis and protein glycosylation, and it participates in various cellular processes. UDP-GlcNAc, a main product of the HBP, functions as a substrate for protein glycosylation and influences various protein functions, especially in cancer cells (Le Minh et al., 2023). O-GlcNAc represents a type of glycosylation, involving the bonding of a lone N-acetylglucosamine (GlcNAc) to a protein’s threonine or serine hydroxyl group *via* an O-glycosidic connection. O-GlcNAc glycosylation is a cyclic process governed by O-GlcNAc hydrolase (OGA) and O-GlcNAc transferase (OGT). OGT catalyzes the transfer of UDP-GlcNAc, adding an O-GlcNAc group to serine or threonine residues on target proteins, while OGA is responsible for removing this modification. Together, they maintain a dynamic equilibrium in the level of this alteration (Parker et al., 2021). O-GlcNAc glycosylation is involved in various biological functions, including transcription, translation, and immune responses (He et al., 2023). It is also strongly associated with tumor cell invasion and metastasis (Packer, 2023), proliferation (You et al., 2023), angiogenesis (Sun et al., 2026), and glucose metabolism (Shi et al., 2022). Additionally, irregular glycosylation of O-GlcNAc is associated with various illnesses, such as metabolic disorders, cancer and cardiovascular disease (Lee et al., 2023). In fact, elevated O-GlcNAc levels or aberrant OGT or OGA expression have been observed in most cancers, such as hepatocellular carcinoma (Tang et al., 2023; Lin et al., 2024), colorectal carcinoma (Cho et al., 2023), lung cancer (Chen et al., 2024), glioblastoma (Leonel et al., 2023), and pancreatic cancer (Motolani et al., 2023), and have been found to promote cancer growth and progression. These findings demonstrate the vital function of O-GlcNAc glycosylation in the development and progression of cancer and other diseases (Banerjee et al., 2016).

Liver kinase B1 (LKBI), which encodes serine-threonine kinase (STK11), is a potent tumor suppressor (Zhang et al., 2024). It was initially found to be mutated in Peutz–Jeghers syndrome (Zhang et al., 2021). Studies have revealed frequent LKB1 mutations in patients with cervical and lung cancers, often in conjunction with KRAS mutations. This correlation is strongly associated with a poor clinical prognosis (Qiao et al., 2024). Lung adenocarcinoma (LUAD) is a malignant tumor that is increasingly receiving global attention, among which LKB1 mutations are a common type in LUAD patients (Angelopoulou et al., 2024). By functioning as an upstream kinase in the AMP-activated protein kinase (AMPK) pathway, LKBI plays a crucial role in maintaining the equilibrium and metabolic processes of cells (Tanaka et al., 2023). LKB1 deactivation leads to an increase in aerobic glycolysis and glucose uptake, inhibiting the regulation of tumor metabolic pathways and thereby contributing to the progression of numerous cancers, including non-small cell lung cancer (Zhang et al., 2015), cervical cancer (Cui et al., 2020), pancreatic cancer (Bildik et al., 2024), and melanoma (Proteau et al., 2023). Activation of the LKB1-AMPK pathway is also closely linked to autophagy, apoptosis, and senescence. However, the regulatory effect of LKB1 on cellular senescence remains incompletely understood. Furthermore, the mechanism by which LKB1 inhibits telomerase activity and whether its action is related to O-GlcNAc glycosylation remain unknown.

Telomere-driven chromosomal instability constitutes a hallmark of LUAD progression. While telomeric DNA inevitably shortens with each somatic division (Thosar et al., 2024), the ribonucleoprotein complex telomerase counteracts this erosion by *de novo* synthesis of (TTAGGG)n repeats (Shay and Wright, 2019), thereby sustaining replicative potential and circumventing senescence. Telomerase is a crucial complex of ribose and nuclear proteins which preserve the structure and length of telomeres (Shay and Wright, 2019). The catalytic core of telomerase is composed of telomerase RNA (TERC) and telomerase reverse transcriptase (TERT), which function cooperatively to maintain telomere length by counteracting telomeric attrition through the synthesis of new telomeric repeats (Shay and Wright, 2019). Telomerase enzymes are absent in somatic cells instead of germ, stem, and embryonic cells possess telomerase activity to maintain telomere length. However, tumor cells have abnormally activated telomerase enzymes to avoid replicative senescence and maintain sustained proliferation (Di Nunno et al., 2023). Aberrant telomerase reactivation is detected in more than 85% LUAD patients, yet the molecular brakes that restrain its activity, particularly the putative involvement of LKB1-dependent O-GlcNAc glycosylation remain undefined. Yang et al. reported that TERT expression is regulated by LKB1 in telomerase-positive LUAD cells (Yang et al., 2017). He et al. also provided evidence that LKB1 effectively suppresses hTERC expression in A549 cells (He et al., 2019). These findings established a significant association between LKB1 and telomerase activity (Tesmer et al., 2023).

There is an immediate need to innovate approaches for LUAD treatment, given that the majority of therapies are ineffective due to the emergence of chemotherapy resistance (Ghanbar and Suresh, 2024). In this research, we investigated the relationships among cellular senescence, telomerase activity, and O-GlcNAc expression in tumor cells in the context of lung cancer treatment. This study represents the first exploration into how O-GlcNAc modification influences telomerase activity regulation. The specific molecular mechanism and upstream regulator LKB1 were subsequently identified *in vitro*. Finally, we used the telomerase inhibitor BIBR1532 and the GFAT inhibitor DON to treat a mouse model established *via* the subcutaneous injection of Lewis Lung Carcinoma Cells to validate treatment efficiency *in vivo*. The findings presented here provide a theoretical basis for the development of clinical treatment strategies for LUAD patients.

## 2 Methods

### 2.1 Cell culture

Human LUAD cells (A549/Tet-LKB1,NCI-H1299-shCtrl,NCI-H1299-shLKB1,NCI-H1975-shCtrl and NCI-H1975-shLKB1) and Lewis Lung Carcinoma Cells (LLC-shCtrl, LLC-shLKB1) were obtained from the lab (Shen et al., 2020) (Liu et al., 2024). And these cells were incubated in Dulbecco’s modified Eagle’s medium (Gibco, USA) supplemented with 10% fetal bovine serum (Atlanta Biologicals), 100 g/mL streptomycin and 100 units/mL penicillin (Gibco, USA). Every cell underwent cultivation at a temperature of 37° C within a humidified atmosphere containing 5% CO2. A549/Tet-LKB1 cells were induced for LKB1 expression *via* tetracycline (Apexbio, Houston, TX, USA). The concentrations of the various drugs used for different cell types in this experiment, along with the treatment durations, are listed in Supplementary Table S1. The sources and catalogue numbers of the drugs used in this study are listed in Supplementary Table S2.

### 2.2 SA-galactosidase activity

The cells from the control and experimental groups were collected and subsequently processed in accordance with the instructions provided by the senescence-galactosidase staining kit (Beyotime, C0602), with the objective of detecting SA-galactosidase activity in the cells.

### 2.3 Real-time quantitative (q)PCR analysis

Our study employed the TRIzol Reagent (Thermo Fisher Scientific) to extract RNA from cells. Reverse transcription (RT)-real-time quantitative (q) PCR (RT-qPCR) analysis was performed to detect human interleukin-1β (IL-1β), CCL5 and SERPINE1 mRNA. GAPDH mRNA detection was used to standardise the RT-qPCR results. Similarly, RT-qPCR was used to detect the mRNA levels of human OGA, OGT, GFAT1, GLUT1 and LKB1, and tubulin mRNA was used as a standardisation control. The primers used in this study are listed in Supplementary Table S3.

### 2.4 Western blot analysis

Protein expression in the control and experimental groups was determined by Western blotting. The ratio of the protein lysis buffer used is RIPA Lysis Buffer (Beyotime):100 mM PMSF (Beyotime): Phosphatase inhibitor (Solarbio) = 100:1:1. The total protein extracts (30–60 µg) were initially separated electrophoretically on 10% SDS‒PAGE gels. The proteins that were visible on the gels were subsequently transferred to nitrocellulose membranes *via* semidry transfer. The antibodies used in this study are listed in the Supplementary Table S4.

### 2.5 Co-immunoprecipitation

Lysis the cells with IP lysis (Beyotime#P0013) buffer under non-denaturing conditions, then add the specific antibody to form a complex with the target antigen, and incubate overnight at 4 °C with gentle agitation. The following day, add 30 μL of Protein A/G (MedChemExpress#HY-P700331A) to the complex and continue incubation for a further 2 h. Wash the magnetic beads with washing buffer, then proceed with gel electrophoresis according to the Western blot protocol outlined above.

### 2.6 EdU incorporation assay

The EdU-555 Cell Proliferation Assay Kit (Beyotime, Shanghai, China) was employed to carry out the EdU assay. A fluorescence microscope (Olympus, Tokyo, Japan) was utilized to observe and capture photographs of the cells. Analysis was performed using ImageJ software; the total number of DAPI-stained cells and the number of EdU-positive cells were counted separately *via* threshold segmentation. After calculating the positivity rate, GraphPad 8.0 software was used to perform tests for statistical significance of differences between groups.

#### 2.6.1 Terminal restriction fragments (TRF) assay

The measurement of telomere length was conducted *via* a Roche kit (Cat.No.12209136001), adhering to the guidelines provided by the manufacturer. TRF analysis was used to evaluate the average length of telomeres. Initially, DNA was isolated from the cells following the TIANGEN kit (Cat# DP304) guidelines, followed by genomic DNA digestion *via* Hinf I and Rsa I and then isolation *via* Southern blotting on a 0.8% agarose gel. Postdenaturation, the gel underwent hybridization using a digoxin-tagged telomere-specific probe (TAGGG) and was identified through an advanced automated chemiluminescence device (Tanon). The average TRF length is calculated based on the chemiluminescence signal.

### 2.7 Telomeric repeat amplification protocol (TRAP)

The activity of telomerase was identified through qPCR *via* the TRAPeze RT Telomerase Detection Kit (Merck, Cat#S7710) in accordance with the guidelines provided by the manufacturer. Quantification of telomerase activity was performed by analysing CT values *via* real-time quantitative PCR (qPCR). Relative activity was calculated by comparing the signal intensity ratio between samples and negative controls during quantification.

### 2.8 Metaphase chromosome segregation and Q-FISH

Treatment of the test and control cells occurred either with drugs or without them for a duration of 24 h, respectively. The acquisition of these cells in the M phase involved a 4-h exposure to colchicine (0.2 μg/mL), succeeded by a 20-min exposure to 75 mM KCl and a minimum 30-min exposure to Carnoy fluid (methanol: glacial acetic acid ratio of 3:1). A droplet of the mixture was applied to a chilled glass slide to break the cell membrane, facilitating chromosome separation. After a flame had passed, the slides were examined under an Olympus oil microscope (BX51, Tokyo, Japan). After the metaphase chromosomes were divided, telomere fluorescence *in situ* hybridization (FISH) was performed. After denaturation at temperatures ranging from 80° C to 85° C, the telomeres underwent hybridization *via* a telomeric PNA probe, specifically in A549/Tet-LKB1 cells. Ultimately, the chromosomes were stained with DAPI and observed *via* confocal microscopy. We calculated the proportion of cases with or without chromosomal abnormalities based on the presence of fluorescent signals from telomeric DNA.

### 2.9 Immunohistochemistry (IHC) assay

Immunohistochemical (IHC) staining was performed using an IHC kit (MXB Bio#KIT-9720). DAB staining was employed using a DAB staining kit (MXB Bio#DAB-0031). The antibodies used in this study are listed in Supplementary Table S3. Quantitative analysis was performed using ImageJ software.

### 2.10 CCK-8 assay

Cells were spread into 96-well plates at a density of 5,000 cells in 100 μL of medium per well, after which different concentrations of drugs were added to treat the cells for 24 h. Cell viability was assayed using the CCK-8 kit (Beyotime, China) according to the manufacturer’s instructions.

### 2.11 Ethics statement and animal experiments

The male C57BL/6 mice were procured from Liaoning Changsheng Biotechnology Co, Ltd. The mice were maintained with food and water provided *ad libitum* at the Animal Experimentation Centre of the College of Basic Medical Sciences, Jilin University (Experimental Animal Use Permit: SCXK (辽)-2020-0001). The animal experiments were conducted in accordance with the ethical standards set out by the Experimental Animal Ethics Committee of Jilin University. C57BL/6 male mice aged between 4 and 5 weeks received injections of the LLC-shCtrl and LLCshLKB1 (1 × 10^6^ cells/mL, 100 μL/mouse). After 1 week, the animals were randomly divided into six groups of six mice each. Mice were then treated i. p. with vehicle, DON (0.8 mg/kg, three times a week), BIBR1532 (1 mg/kg, three times a week), or with combinations. PBS was used as an isotype control vehicle for DON and BIBR1532. The mice’s weight and the tumors’ width and length were assessed on a daily basis. The tumor volume (μL) was calculated as width^2^ × length × 0.5. Following 3 weeks of the treatment, during which the maximum tumor volume reached 1,300 mm^3^, the mice were humanely euthanized by inhalation of CO_2_. The tumors were then eviscerated and weighed, and the volume was calculated. The lungs, hearts, kidneys, livers, and spleens were subsequently collected for further analysis. Every tissue sample was preserved in formalin, encased in paraffin, sliced, and dyed using haematoxylin and eosin (H&E), and subjected to immunohistochemical (IHC) analysis. All the experiments were conducted in accordance with the relevant guidelines and regulatory frameworks.

### 2.12 Statistical analysis

GraphPad Prism 8.0.1 (GraphPad Software, California, USA) was used to perform statistical analyses. Experiments were repeated at least three times and analysed using ImageJ. Datas are presented as means ± SEM. Statistical signiffcance was determined using Student’s t-test and analysis of variance (ANOVA). *p* values less than or equal to 0.05 was considered statistically significant. All quantitative data were collected from three independent biological replicates (n = 3). *p < 0.05, **p < 0.01, ***p < 0.001, and ****p < 0.0001 indicated statistical significance. All quantitative data were collected from three independent biological replicates (n = 3). All exact p-values for the statistical tests in this study are summarised in Supplementary Table S5; only the significance intervals are indicated in the main text, and the detailed values can be found in this Supplementary Table S5.

## 3 Results

### 3.1 LKB1 inhibits O-GlcNAc glycosylation in lung adenocarcinoma

As the main regulator of glucose metabolism, LKB1 stimulates gene expression linked to crucial enzymes in various metabolic processes, such as aerobic oxidation, gluconeogenesis, glycolysis and the pentose phosphate pathway (Tanaka et al., 2023). Therefore, we constructed an A549/Tet-LKB1 cell line that can induce LKB1 expression *via* tetracycline, and we demonstrated that treatment with 1 µM tetracycline for 24h can effectively induce LKB1 expression as previously reported (Shen et al., 2020). We selected the LKB1 mutant lung adenocarcinoma A549 cell line as the research model to observe the effect of LKB1 on O-GlcNAc glycosylation. Besides, we also selected the LKB1 wild-type lung adenocarcinoma cell line NCI-H1975, and conducted further experiments involving LKB1 knockdown. Control cells were transfected with an empty vector, while experimental groups were transfected with plasmids to knock down LKB1, thereby establishing LKB1-knockdown tumor cell lines. Western blot was conducted in these two different cell lines to assess O-GLcNAc related protein expression. The results showed that the whole level of O-GlcNAc which labeled by RL2 antibody was significance decreased after LKB1 re-expressed in A549 cells (Figures 1A,B), while the knockdown in H1975 showed obviously increased (Figures 1C,D). In addition, the effects of LKB1 on GFAT1, a key enzyme in the hexosamine biosynthetic pathway (HBP); GLUT1, the transporter that regulates glucose entry into the HBP; and OGA and OGT, enzymes involved in the regulation of O-GlcNAc modification, were assessed by Western blot analysis. Tetracycline-induced LKB1 expression increased LKB1 and GLUT1 protein expression. Nevertheless, in A549/Tet-LKB1 cells, no notable alterations in OGA,OGT or GFAT1 protein expression were observed in comparison with those in the control group (Figures 1A,B). In H1975 cells, the differences in OGA,OGT and GFAT1 expression were not statistically significant, whereas LKB1 expression was significantly decreased (Figures 1C,D) and GLUT1 expression was elevated. We also selected another non-small cell lung cancer to assess the influence of LKB1 on O-GlcNAc level. Western blot analysis showed that after LKB1 knockdown the whole O-GLcNAc level were significantly increased which same as the results in H1975 cells (Supplementary Figure S1A). In H1299 cells, OGT and GLUT1 were elevated, with no significant change in GFAT1 and a significant decrease in LKB1 (Supplementary Figure S1B). After LKB1 was knocked down in H1975 and H1299 cells, GLUT1 did not show the opposite trend to that after the induction of LKB1 expression in A549/Tet-LKB1 cells, which could be due to differences in the cell source or the different basal levels of GLUT1 in different cells, leading to inconsistent trends (Chen et al., 2019). Furthermore, the mRNA levels of OGA, OGT, GFAT1, GLUT1, and LKB1 were detected in A549/Tet-LKB1 and H1975 cells using quantitative PCR (q-PCR). The results demonstrated that when LKB1 was overexpressed using tetracycline induction, there were no significant differences in the mRNA levels of OGA, OGT, and GFAT1; however, the mRNA levels of GLUT1 and LKB1 were significantly increased. In H1975 cells, following LKB1 knockdown, a significant reduction in LKB1 mRNA levels was observed, while OGA, OGT, and GFAT1 mRNA levels remained unchanged. The mRNA levels of GLUT1 exhibited a slight increase (Figures 1E,F). These findings suggest that LKB1 significantly reduces overall O-GlcNAc glycosylation in LUAD cell lines.

**FIGURE 1:**
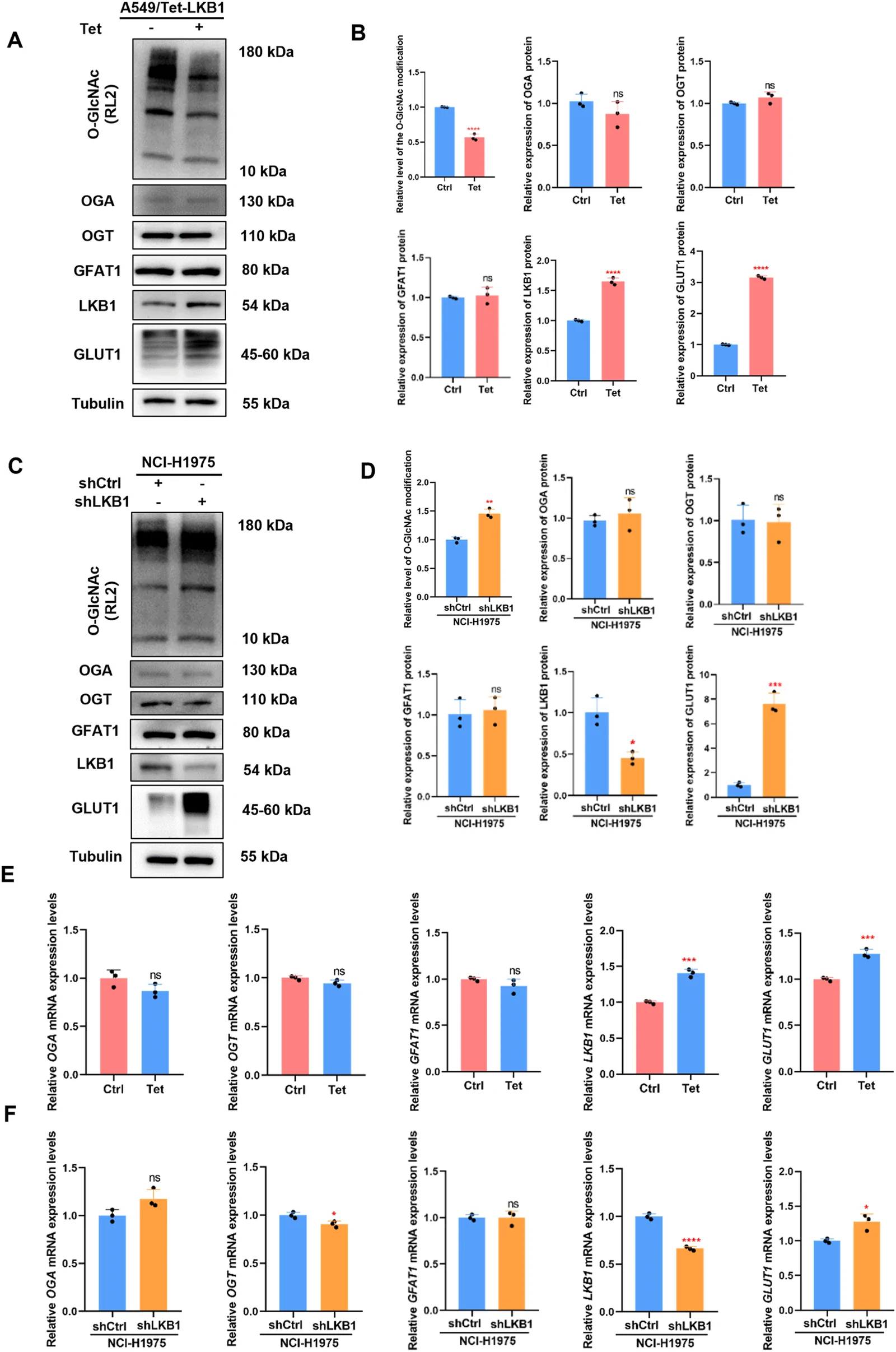
LKB1 inhibits O-GlcNAc glycosylation in lung adenocarcinoma **(A,B)** Western blotting to detect the protein expression levels of O-GlcNAc modification, OGA, OGT, GFAT1, LKB1, GLUT1, and Tubulin in A549/Tet-LKB1 cells and graphs of the statistical analysis (normalized to Tubulin). **(C,D)** Western blotting to detect the protein expression levels of O-GlcNAc modification, OGA, OGT, GFAT1, LKB1, GLUT1, and Tubulin in NCI-H1975 cells and graphs of the statistical analysis (normalized to Tubulin). **(E,F)** q-PCR assays to detect the mRNA expression levels of *OGA, OGT, GFAT1, LKB1 and GLUT1* in A549/Tet-LKB1 cells **(E)** and NCI-H1975 cells (normalized to Control) **(F)** (n = 3, ns, *P* > 0.1, no significant difference; *, *P* < 0.05; ***, *P* < 0.001; ****, *P* < 0.0001).

### 3.2 O-GlcNAc glycosylation inhibits cellular senescence in lung adenocarcinoma

According to the key roles in O-GlcNAc regulation, a competitive inhibitor of OGT (OSMI-1) and an OGA inhibitor (Thiamet-G) were selected as treatment agents to inhibit or activate O-GlcNAc in our research. In order to ascertain the most efficacious drug concentration, CCK8 experiments were conducted in order to detect the IC50 values of OSMI-1 and Thiamet-G in A549/Tet-LKB1, NCI-H1975 and NCI-H1299 cells (Supplementary Figures S2A-F). In accordance with the established IC50 values and in consideration of the pertinent literature reports, a series of Western blot experiments were conducted at varying concentrations (Sun et al., 2020) (Supplementary Figures S3A-F). For subsequent experiments, we ultimately decided to treat all cells with 5 µM OSMI-1 and 10 µM Thiamet-G for 24 h (Supplementary Table S1).

Senescence prevents the replication of damaged genomes and therefore has antitumor effects. However, owing to the epigenetic similarities between senescent cells and tumor cells, the repression of certain tumor suppressors or the activation of oncogenes within senescent cells could promote tumorigenesis; consequently, cellular senescence plays a dual role in tumorigenesis (Colucci et al., 2024). We subsequently investigated the effect of O-GlcNAc glycosylation on tumor cell senescence in lung adenocarcinoma cells. As SA-β-gal staining is the gold standard for detecting cell senescence level, we firstly quantified the number of senescent cells in A549/Tet-LKB1-Ctrl group and H1975 group after O-GlcNAc glycosylation was inhibited or promoted. The results showed that the inhibition of O-GlcNAc glycosylation with OSMI-1 resulted in an increased number of senescent tumor cells. Conversely, the addition of thiamet-G promoted O-GlcNAc glycosylation, leading to a decreased number of senescent tumor cells, both in A549/Tet-LKB1-Ctrl group and H1975 group (Figures 2A,B). We subsequently assessed senescence-related markers *via* Western blotting in A549/Tet-LKB1 cells; OSMI-1-mediated inhibition of O-GlcNAc glycosylation significantly increased the protein expression of RB, p53, and p21, whereas P-RB protein expression was reduced compared with that in the control group. Furthermore, the ratio of phosphorylated RB to total RB indicates that, following inhibition of O-GlcNAc glycosylation by OSMI-1, RB shifts towards a hypophosphorylated form associated with cellular senescence (Figure 2C). In contrast, thiamet-G induced an increase in O-GlcNAc, yielding the opposite outcome. However, neither drug influenced the expression level of the apoptotic protein BAX (Figure 2C). We then evaluated senescence-associated markers *via* Western blotting in H1299 cells harboring a p53 mutation. Compared with the control, the inhibition of O-GlcNAc glycosylation resulted in a significant increase in RB and p21 protein expression, along with a decrease in P-RB protein expression. The ratio of phosphorylated RB to total RB indicates that, following the inhibition of O-GlcNAc glycosylation by OSMI-1, the P-RB/RB ratio decreases, suggesting an increase in cellular senescence. Conversely, an increase in O-GlcNAc levels elicited the opposite effects. However, neither drug altered the expression of the apoptotic protein BAX (Supplementary Figure S4A).

**FIGURE 2:**
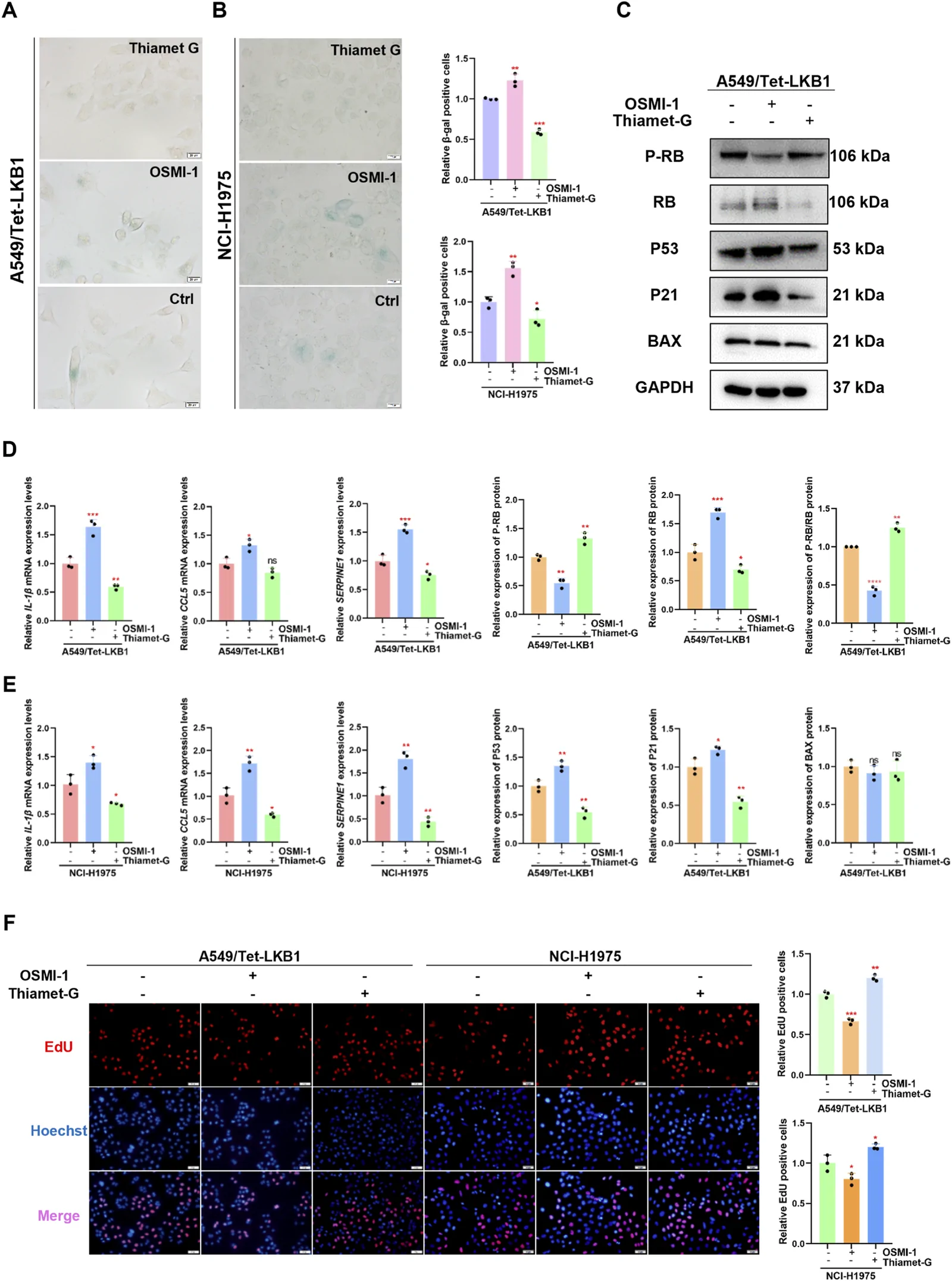
O-GlcNAc glycosylation inhibits cellular senescence in lung adenocarcinoma A-B. β-Galactosidase assays were used to detect senescence in A549/Tet-LKB1 cells **(A)** and NCI-H1975 cells **(B)** (scale bar, 50 μm), and graphs of the statistical analysis are shown (normalized to Control). **(C)** Western blotting was used to detect p53, p21, RB, P-RB, BAX, and GAPDH protein expression in A549/Tet-LKB1 cells, and the results were statistically analyzed (normalized to GAPDH). **(D,E)** qPCR detection of the mRNA expression of *IL-1β*, *CCL5*, and S*ERPINE1* in A549/Tet-LKB1 cells **(D)** and NCI-H1975 cells **(E)** (normalized to Control)**. (F)** EdU assay to detect A549/Tet-LKB1 and NCI-H1975 cell proliferation (scale bar, 20 μm) and graphs of the statistical analysis (normalized to Control) (n = 3, ns, *P* > 0.1, no statistically significant difference; *, *P* < 0.05; **, *P* < 0.01; ***, *P* < 0.001).

Senescent cells generate SASP, a senescence-associated secretory phenotype that can alter the extracellular matrix composition, impair stem cell functions, promote cellular transdifferentiation, and induce a senescent phenotype in surrounding cells, resulting in systemic chronic inflammation (Okawa et al., 2024). Senescent cells exhibiting the SASP commonly express a range of markers, such as matrix metalloproteinases (MMP3, MMP9), interleukins (IL-1α, IL-1β, and IL-6), chemokines (CCL2, CCL20, CCL5, CXCL2, and CXCL5), and SERPINE1 (Li et al., 2024). To elucidate the impact of O-GlcNAc modifications on senescence, we conducted qPCR analyses on A549/Tet-LKB1 (Figure 2D) and H1975 cells (Figure 2E) to evaluate the mRNA expression of SASP factors. OSMI-1 elevated the expression levels of *IL-1β*, *CCL5*, and *SERPINE1* relative to the control levels. Conversely, thiamet-G decreased the expression of *IL-1β*, *SERPINE1*, and *CCL5* in H1975 cells but had no significant effect on *CCL5* expression in A549/Tet-LKB1 cells. A lack of DNA replication is also a sign that senescence is initiating. Therefore, an EdU assay was performed. EdU was utilized to assess tumor cell proliferation. We similarly performed an assay on A549/Tet-LKB1 and H1975 cells following treatment with drugs designed to inhibit and promote O-GlcNAc glycosylation, respectively. Cell proliferation was reduced following OSMI-1-induced inhibition of O-GlcNAc glycosylation, whereas it was enhanced with thiamet-G-mediated promotion of O-GlcNAc glycosylation (Figure 2F). These findings suggest that inhibiting O-GlcNAc glycosylation may promote tumor cell senescence and hinder DNA Livereplication in lung adenocarcinoma.

### 3.3 Inhibition of O-GlcNAc glycosylation reduces telomerase activity and induces telomere-associated chromosome dysfunction

In order to ascertain the role played by O-GlcNAc modifications in telomerase regulation, we first monitored changes in telomerase activity after inducing or inhibiting O-GlcNAc modifications in A549 cells. Telomerase activity was measured using the telomeric repeat amplification protocol (TRAP), and telomere length was determined *via* the telomere restriction fragment (TRF) assay. The results showed that OSMI-1 reduces telomerase activity and telomere length, whereas thiamet-G enhances O-GlcNAc glycosylation, increases telomerase activity and elongates telomeres (Figures 3A–C). Chromosomal instability arises when telomeres shorten to a critical length. Telomere-associated chromosomal dysfunction mainly includes telomere fusion, multiple telomere signaling (MTS), and telomere deletion (Lee et al., 2023). To determine whether telomere-associated chromosomal dysfunction is responsible for the effect of O-GlcNAc glycosylation on telomerase, we performed quantitative fluorescence *in situ* hybridization (Q-FISH) experiments to characterize the telomere structure *via* a PNA probe, which is visible *via* fluorescence microscopy. The results showed that the inhibition of O-GlcNAc glycosylation by OSMI-1 led to increased levels of telomere deletion (quantified by % of signal-free ends), fusion and increased telomere signaling (quantified by MTS numbers) compared with those in the control group of A549 cells. In contrast, the addition of thiamet-G, which stimulates O-GlcNAc glycosylation, reversed these effects (Figures 3D–G). Immunoprecipitation assays further confirmed that TERT is modified by O-GlcNAc glycosylation (Supplementary Figure S4B). These results demonstrate a significant correlation between O-GlcNAc modification level and telomerase activity, which suggests a potential regulatory relationship between them.

**FIGURE 3:**
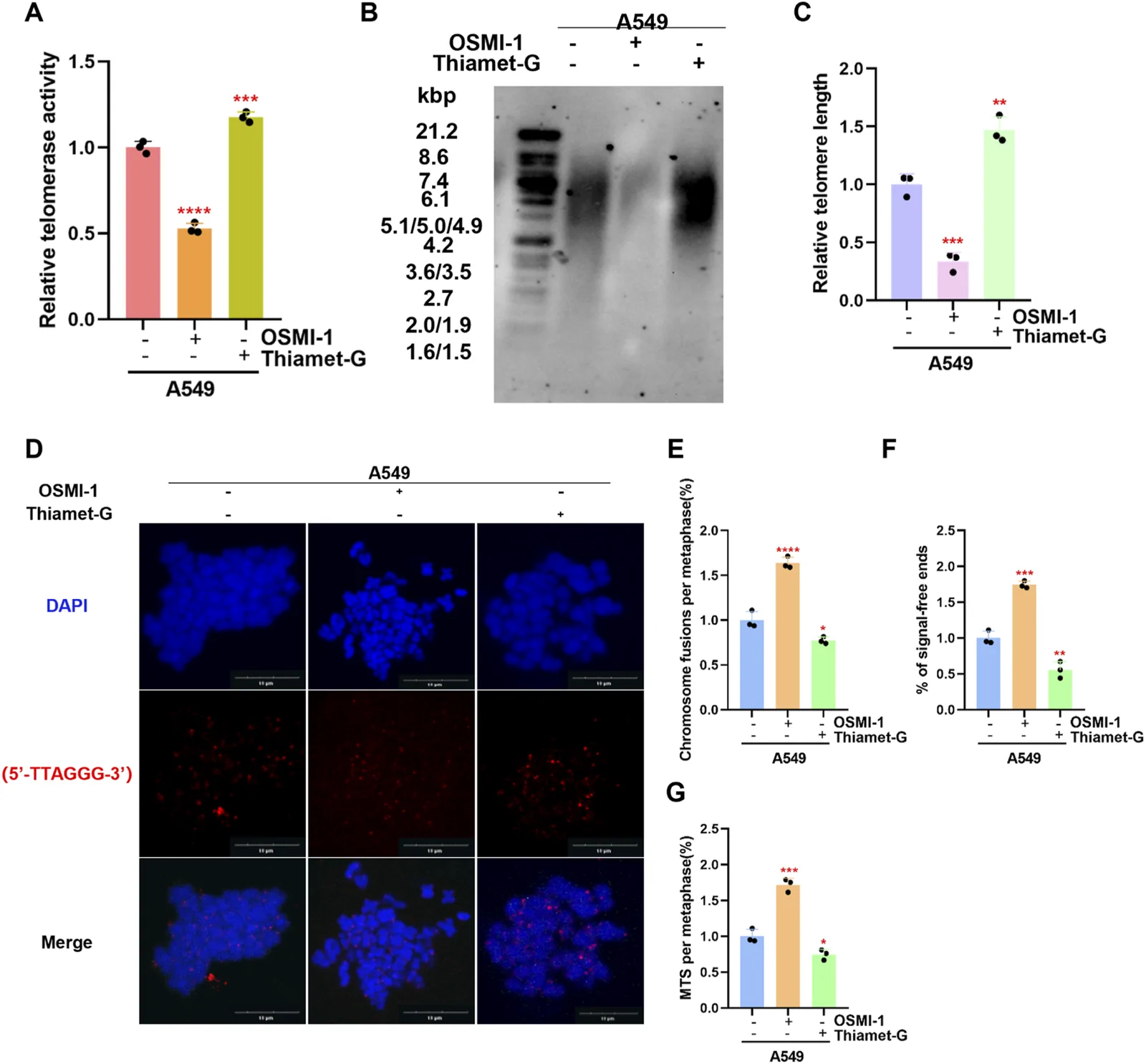
Inhibition of O-GlcNAc glycosylation reduces telomerase activity and induces telomere-associated chromosome dysfunction **(A)** Telomeric repeat amplification protocol (TRAP) assay for telomerase activity. **(B,C)** Telomere restriction fragment (TRF) assay for telomere length and graphs of the statistical analysis (kbp unit is used to characterize the length of telomeric DNA fragments). **(D-G)** Quantitative fluorescence *in situ* hybridization (Q-FISH) to detect chromosomal aberrations (scale bar, 10 μm) and graphs of statistical analysis (normalized to Control) (n = 3, *, *P* < 0.05; **, *P* < 0.01; ***, *P* < 0.001; ****, *P* < 0.0001).

### 3.4 LKB1 promotes cellular senescence by regulating HBP to inhibit telomerase activity

We subsequently examined whether LKB1 regulates tumor cell senescence and telomerase activity through HBP. Firstly, the overall levels of O-GlcNAc modification in A549/Tet-LKB1 and NCI-H1975 cells were determined by means of Western blot analysis. In comparison with the control group, tetracycline-induced LKB1 expression significantly reduced the levels of O-GlcNAc modification. In contrast, co-treatment with tetracycline and the O-GlcNAcylation enhancer thiamet-G resulted in the restoration of O-GlcNAc levels. Meanwhile, inhibition of O-GlcNAcylation with OSMI-1 resulted in reduced O-GlcNAc levels. Correspondingly, in H1975 cells, both LKB1 knockdown and thiamet-G treatment elevated cellular O-GlcNAc levels. Furthermore, treatment with OSMI-1 effectively reduced the elevated O-GlcNAc levels resulting from LKB1 knockdown (Figure 4A). These results confirm the efficacy of OSMI-1 and Thiamet-G in regulating O-GlcNAc levels and validate their utility as reliable tools for further research in this field.

**FIGURE 4:**
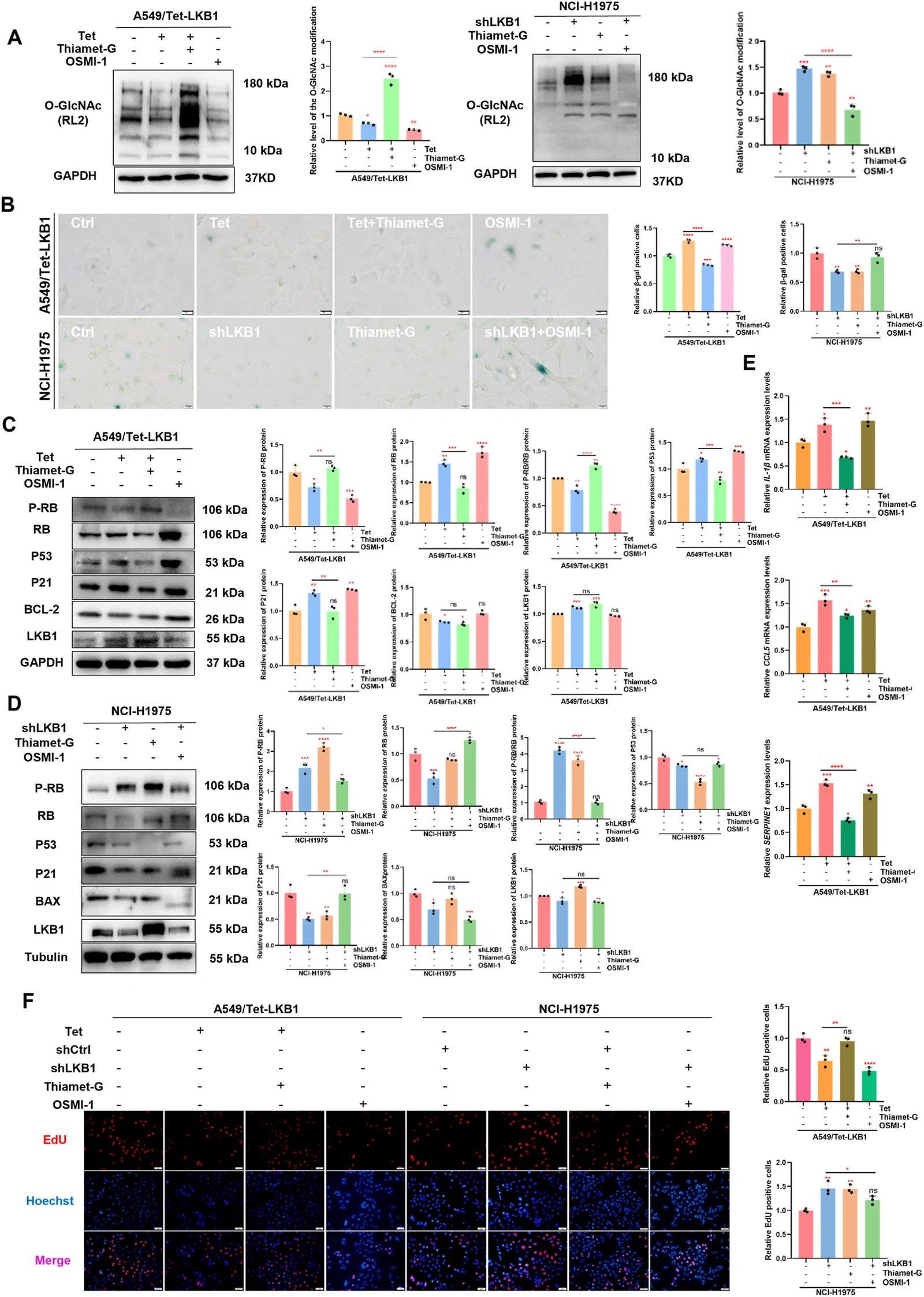
LKB1-mediated inhibition of O-GlcNAcylation promotes cellular senescence and attenuates DNA replication **(A)** Western blot detection of O-GlcNAc modification and GAPDH in A549/Tet-LKB1 cells and NCI-H1975 cells and statistical analysis of the data (normalized to GAPDH). **(B)** β-Galactosidase assay was used to detect senescence in A549/Tet-LKB1 cells and NCI-H1975 cells (scale bar, 50 μm), and the results were statistically analyzed (normalized to Control). **(C,D)** Western blot detection of P-RB, RB, p53, p21, BCL-2, BAX and GAPDH protein expression levels in A549/Tet-LKB1 cells,NCI-H1975 cells, and statistical analysis of the data (normalized to GAPDH). **(E)** qPCR was used to detect the SASP-related mRNAs in A549/Tet-LKB1 cells (normalized to Control). **(F)** EdU assay for the detection of cell proliferation in A549/Tet-LKB1 cells and NCI-H1975 cells (scale bar, 20 μm) and plot of the statistical analysis (n = 3, ns, *P* > 0.1, no significant difference; *, *P* < 0.05; **, *P* < 0.01; ***, *P* < 0.001; ****, *P* < 0.0001).

To further investigate the senescent phenotype, we examined the expression of key senescence-associated markers. SA-β-gal staining showed that tetracycline-driven LKB1 expression sharply elevated β-gal positive A549/Tet-LKB1 cells. And this rise was reversed by co-administration of thiamet-G, whereas OSMI-1-mediated blockade of O-GlcNAcylation further augmented senescence. Similarly, SA-β-Gal assays revealed that LKB1 knockdown or Thiamet-G mediated O-GlcNAcylation both reduced the senescent fraction, whereas OSMI-1-induced inhibition of O-GlcNAc increased it, indicating that LKB1 drives senescence *via* modulation of O-GlcNAc glycosylation (Figure 4B). We next evaluated the expression levels of senescence-associated proteins. In A549/Tet-LKB1 cells, tetracycline treatment upregulated the protein levels of LKB1, RB, p53, and p21, accompanied by RB dephosphorylation and slightly inhibiting BCL-2, relative to the vehicle control. These senescence-like alterations were largely reversed by co-administration of thiamet-G, with the exception of LKB1 and BCL-2 protein levels, which remained unchanged. In contrast, selective inhibition of O-GlcNAcylation with OSMI-1 reproduced the tetracycline-induced phenotype, characterized by increased RB, p53, and p21, along with decreased phosphorylated RB, but did not alter BCL-2 levels or apoptotic indices. Furthermore, the ratio of phosphorylated RB to total RB is consistent with the trend in P-RB expression (Figure 4C). Western blot analysis demonstrated that LKB1 knockdown in H1975 cells reduced the expression of LKB1, RB, p21, and BAX, elevated phosphorylated RB levels. Enhancement of O-GlcNAcylation by thiamet-G further increased phosphorylated RB levels, while attenuating p53 and p21, without affecting RB and BAX. Conversely, pharmacological inhibition of O-GlcNAcylation with OSMI-1 reversed the LKB1-knockdown signature, leading to a decrease in phosphorylated RB and increases in RB, p53, and p21; BAX remained unaltered, confirming that OSMI-1 does not engage apoptotic pathways. The phosphorylated RB to total RB ratio was consistent with the trend in P-RB expression (Figure 4D). We also assessed the expression of senescence-associated markers at the mRNA level by qPCR. The results indicated that tetracycline treatment significantly upregulated the mRNA expression of *IL-1β*, *CCL5*, and *SERPINE1* compared with the vehicle control. In contrast, these increases were attenuated when cells were co-treated with thiamet-G. Similarly, administration of OSMI-1 alone also elevated the mRNA levels of *IL-1β*, *CCL5*, and *SERPINE1* (Figure 4E). DNA synthesis capacity was next evaluated by EdU incorporation assay. The results showed that treatment with tetracycline significantly reduced the proportion of EdU-positive cells relative to the control. This suppression of DNA replication was mitigated by co-administration of thiamet-G. Conversely, OSMI-1 alone decreased the EdU-positive cell population. In H1975 cells, EdU assays indicated that both LKB1 knockdown and the addition of Thiamet-G induced enhanced tumor cell proliferation, suggesting that O-GlcNAcylation promotes tumour cell growth. In contrast, OSMI-1-mediated inhibition of O-GlcNAcylation suppressed proliferation in LKB1-deficient cells (Figure 4F). These findings indicate that LKB1-mediated inhibition of O-GlcNAcylation promotes cellular senescence and attenuates DNA replication.

To elucidate how LKB1 suppresses telomerase activity *via* the HBP pathway, we analyzed A549/Tet-LKB1 and H1975 cells by measuring telomerase activity (TRAP), telomere length (TRF), and telomere-associated chromosome dysfunction (Q-FISH). TRAP assays demonstrated that telomerase activity was reduced upon LKB1 induction or OSMI-1 treatment, an effect that was reversed by the addition of both Tet and Thiamet-G in A549/Tet-LKB1 cells (Figure 5A). Similarly, in H1975 cells, telomerase activity was significantly enhanced by LKB1 knockdown or Thiamet-G treatment. This enhanced activity was attenuated by OSMI-1 administration, even in the context of LKB1 knockdown (Figure 5B). The telomere length data corroborated the telomerase activity findings. Tetracycline-induced LKB1 expression shortened telomeres, and this effect was rescued by Thiamet-G. In contrast, inhibition of O-GlcNAcylation with OSMI-1 alone was sufficient to shorten telomeres. These results showed that O-GlcNAcylation is crucial for telomere maintenance downstream of LKB1 (Figures 5C,D). Addition of Thiamet-G reversed the effects induced by Tet-mediated LKB1 overexpression, including telomere deletion, fusion, and the increase in multiple telomere signaling events. In contrast, treatment with OSMI-1 alone produced effects similar to LKB1 overexpression (Figure 5E). These findings suggest that LKB1 inhibits O-GlcNAc glycosylation, suppresses telomerase activity, and reduces telomere length. To further dissect the clinical implications of LKB1 mutations in LUAD and clarify the regulatory association between LKB1 and core components of the O-GlcNAc signaling pathway as well as telomerase-related genes, we performed Gene Set Enrichment Analysis (GSEA) on the publicly available GSE51266 dataset to evaluate pathway-specific enrichment patterns under the LKB1-knockout genetic background. GSEA plots demonstrated statistically significant shifts in normalized enrichment scores for the predefined gene sets of O-GlcNAc biosynthesis and telomere maintenance, indicating that the transcriptional programs governing these two biological processes are profoundly and differentially modulated upon LKB1 depletion (Supplementary Figure S4C). Consistent with this observation, unsupervised hierarchical clustering heatmaps were constructed to visualize the distinct transcriptional landscapes of O-GlcNAc biosynthesis- and telomere maintenance-related genes between the control and LKB1-knockdown groups. The heatmap profiling of key O-GlcNAc pathway components (including B3GLCT, OGT, LFNG, and POFUT2, *etc.*) exhibited a clearly separated expression signature after LKB1 knockdown. Meanwhile, the heatmap of telomerase-associated gene (such as STN1, SLX4, RAD50, and POT1, *etc.*) also displayed a markedly divergent expression profile between the two experimental conditions (Supplementary Figure S4D). Collectively, these integrated transcriptomic analyses provide orthogonal bioinformatic evidence supporting a previously underappreciated regulatory role of LKB1 in governing both O-GlcNAc biosynthesis and telomere maintenance pathways in the LUAD context.

**FIGURE 5:**
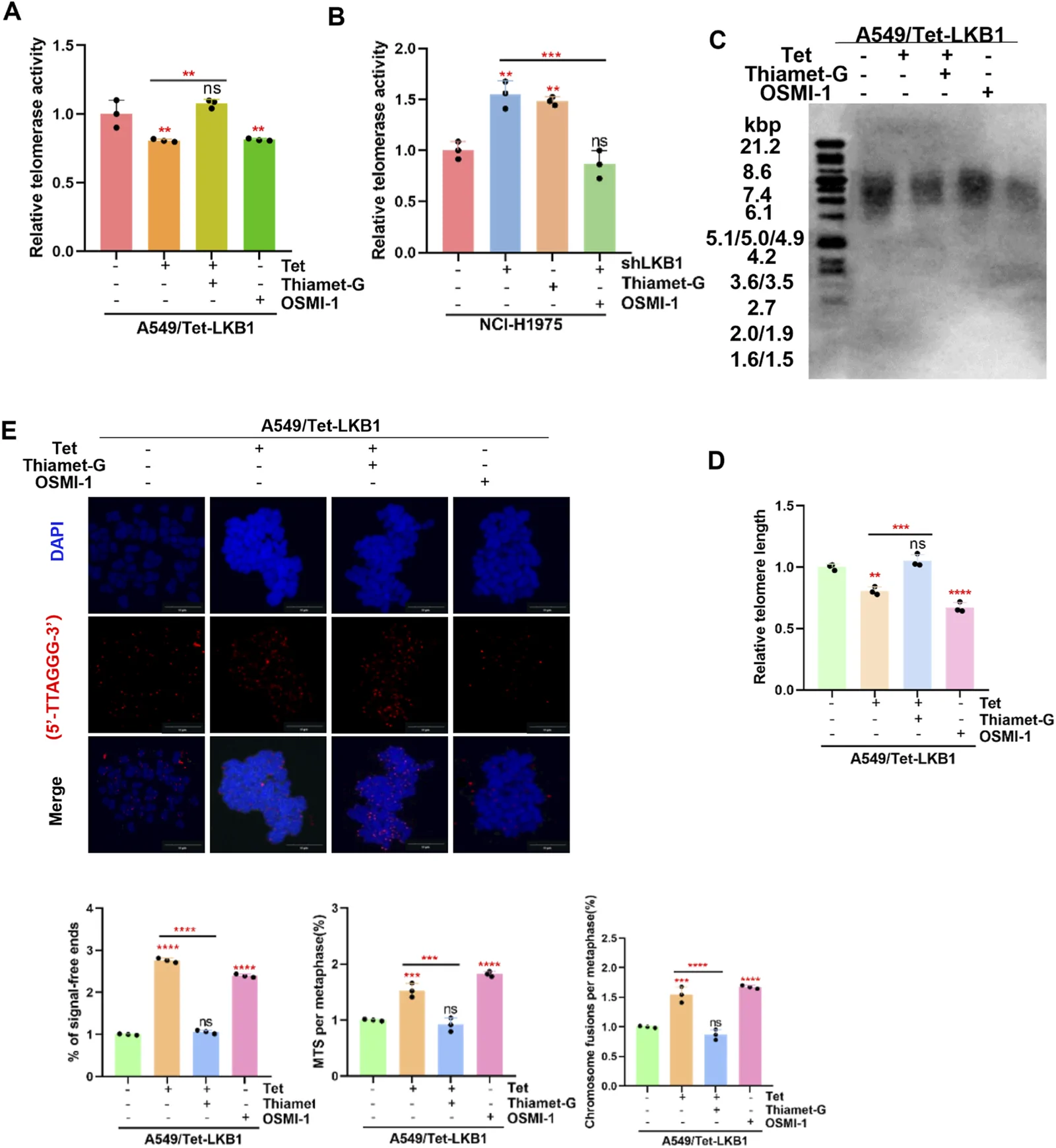
LKB1 inhibits O-GlcNAc glycosylation, suppresses telomerase activity, and reduces telomere length. **(A,B)** Telomeric repeat amplification (TRAP) assay to determine telomerase activity in A549/Tet-LKB1 cells and NCI-H1975 cells. **(C,D)** Telomere restriction fragment (TRF) assay to determine telomere lengths in A549/Tet-LKB1 cells and graphs of the statistical analysis (kbp unit is used to characterize the length of telomeric DNA fragments). **(E)** Quantitative fluorescence *in situ* hybridization (Q-FISH) assay to detect chromosomal aberrations (scale bar, 20 μm) and graphs of the results of the statistical analysis (normalized to Control) (n = 3, ns, *P* > 0.1, no significant difference; **, *P* < 0.01; ***, *P* < 0.001; ****, *P* < 0.0001).

### 3.5 AMPK-related phosphorylation of GFAT1 participates in HBP regulation *via* LKB1

AMPK is a key downstream target and effector of LKB1 (Hollstein et al., 2019). To investigate whether AMPK is responsible for the LKB1-mediated regulation of O-GlcNAc glycosylation, we first used AMPK inhibitors and activators to treat A549/Tet-LKB1 cells and then performed Western blotting. The results showed that concurrent restoration of LKB1 expression and inhibition of AMPK with Compound C led to the recovery of global O-GlcNAc glycosylation levels. Conversely, activation of AMPK (with A-769662) reduced global O-GlcNAc glycosylation levels (Figures 6A,B). These findings suggest that the LKB1–AMPK axis negatively regulates O-GlcNAc glycosylation in A549 cells. Zibrova et al. demonstrated that vascular endothelial growth factor inhibits its activity through the AMPK-mediated phosphorylation of GFAT1, thereby mitigating the inhibitory effect of the HBP on angiogenesis (Zibrova et al., 2017). Prior work has established AMPK as a kinase capable of phosphorylating GFAT1 (Zibrova et al., 2017), and GFAT1 plays a key role in regulating metabolism as the rate-limiting enzyme in the HBP (Wei et al., 2024). Based on these published observations, we hypothesized that AMPK-mediated GFAT1 phosphorylation may modulate HBP flux, and next investigated whether GFAT1 is involved in the regulatory effect of LKB1 on the HBP. Western blotting was used to determine the protein expression of LKB1, p-AMPK, AMPK, and GFAT1. The results revealed no significant changes in the levels of AMPK and GFAT1 compared with those in the control group. Additionally, the addition of tetracycline induced LKB1 expression, whereas the combination of tetracycline and Compound C inhibited p-AMPK. Furthermore, the addition of A-769662 activated p-AMPK. The p-AMPK to AMPK ratio showed a trend consistent with that of p-AMPK expression (Figure 6C). Subsequently, to investigate whether LKB1-AMPK affects telomerase activity, we employed the TRAP assay. In A549/Tet-LKB1 cells, tetracycline was added to induce LKB1 expression, while Compound C was used to inhibit AMPK concurrently with LKB1 induction. Results demonstrated that AMPK inhibition restored LKB1’s suppression of telomerase activity. Additionally, AMPK inhibitor (Compound C) and activator (A-769662) were separately administered to A549/Tet-LKB1 cells. Results demonstrated that compared to the control group, AMPK inhibition led to increased telomerase activity in tumor cells, while AMPK activation resulted in decreased telomerase activity (Figure 6D). These results demonstrate that AMPK participates in the LKB1-mediated regulation of telomerase. It was reported that AMPK inhibition by cisplatin reduces GFAT1 phosphorylation, leading to enhanced GFAT1 enzymatic activity (Wang et al., 2021). Based on our experimental findings demonstrating unaltered GFAT1 protein expression levels, we postulate that AMPK-mediated regulation occurs primarily through modulation of GFAT1 phosphorylation status rather than through transcriptional or translational control. Through coimmunoprecipitation (co-IP) assays in A549 cells, we detected phosphorylated GFAT1 signal (Figure 6E). Furthermore, our co-immunoprecipitation experiments provide direct biochemical evidence for a physical interaction between AMPK and GFAT1 (Figure 6F). Collectively, the above findings indicate that the LKB1–AMPK signaling acts on GFAT1 through a direct protein interaction and may exert further effects by modulating its phosphorylation state. However, the direct, site-specific phosphorylation of GFAT1 by AMPK at defined residues remains to be experimentally validated in future studies.

**FIGURE 6:**
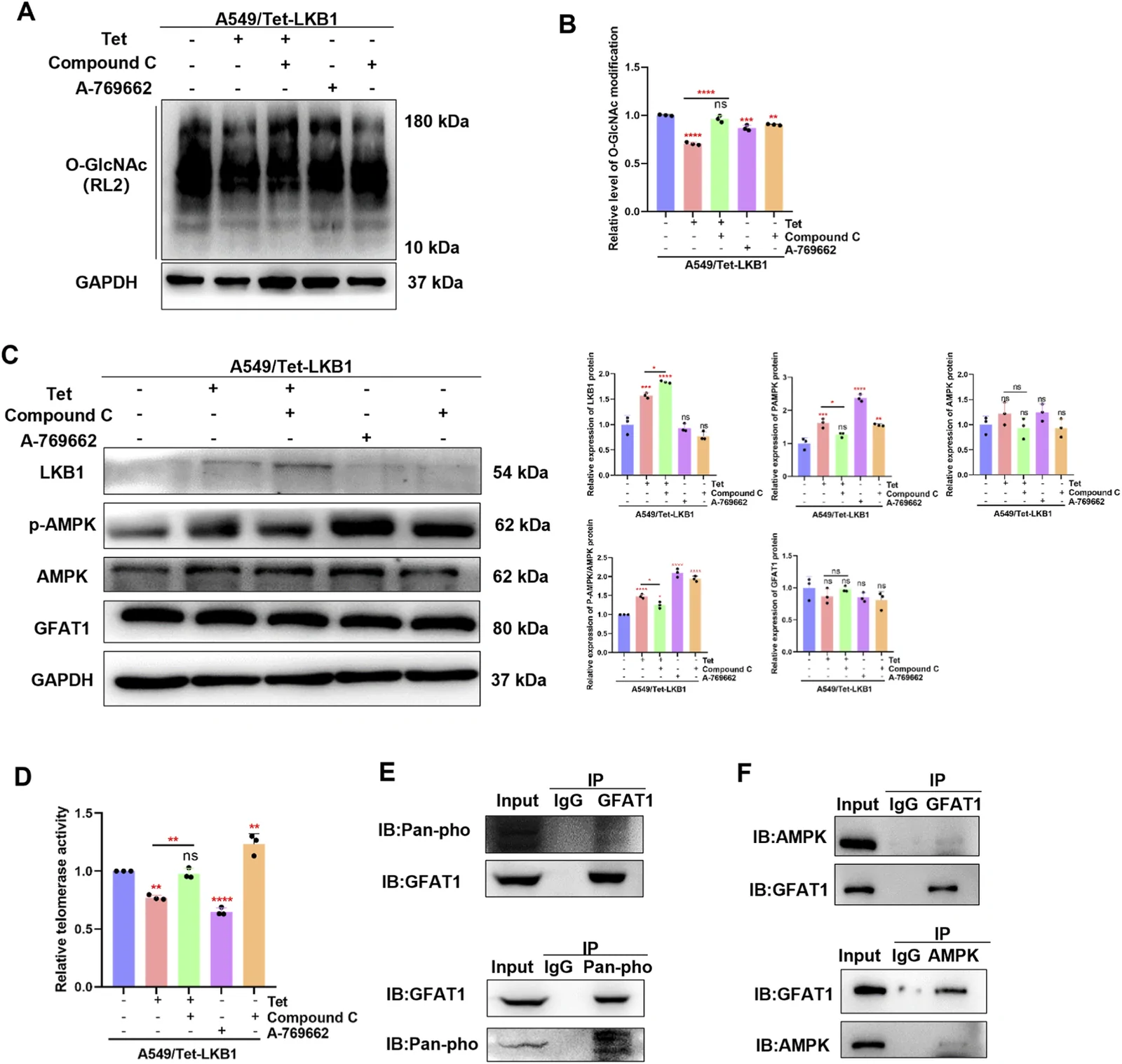
AMPK-related phosphorylation of GFAT1 participates in HBP regulation *via* LKB1 **(A,B)** Western blotting was used to detect the protein expression levels of O-GlcNAc modification and GAPDH, and graphs of the results of the statistical analysis are shown (normalized to GAPDH). **(C)** Western blotting was used to detect the protein expression levels of LKB1, p-AMPK, AMPK, GFAT1, and GAPDH, and graphs of the statistical analysis results are shown (normalized to GAPDH). **(D)** Telomeric repeat amplification (TRAP) assay to determine telomerase activity in A549/Tet-LKB1 cells. **(E,F)** Co-immunoprecipitation assays revealed changes in the phosphorylation status of GFAT1 and demonstrated that AMPK interacts with GFAT1 in A549 cells. (ns, *P* > 0.1, no statistically significant difference; *, *P* < 0.05; **, *P* < 0.01; ***, *P* < 0.001; ****, *P* < 0.0001).

### 3.6 Therapeutic efficacy of O-GlcNAcylation-targeted intervention in lung adenocarcinoma

To assess the *in vivo* tumor-suppressive effects of the GFAT inhibitor DON and the telomerase inhibitor BIBR1532, we applied various drug regimens to a subcutaneous tumor xenograft model in C57BL/6 mice, which was established with LLC-shCtrl and LLC-shLKB1 cells. The drug administration regimen is depicted in Figure 7A. The results of the immunohistochemistry (IHC) experiments performed to validate the efficiency of LKB1 knockdown are shown in Supplementary Figure S5A. In mice treated with BIBR1532, tumor growth was partially inhibited compared with that in the LLC-shCtrl group, whereas mice in the LLC-shLKB1 group had significantly enlarged tumors. Tumor growth in mice treated with either BIBR1532 or DON monotherapy was reduced relative to that in the LLC-shLKB1 group. Moreover, combination treatment with these two agents resulted in the most significant tumor inhibition. These findings indicate that LKB1 deficiency is associated with substantial tumor growth, which can be mitigated by BIBR1532 and DON administration (Figures 7B–D). IHC staining results showed that compared with the LLC-shCtrl group, the expression levels of p53 were elevated, and those of PCNA were reduced in the tumor tissues of mice in the LLC-shCtrl + BIBR1532 group, while there were no significant differences in the expression levels of BCL-2, Caspase3 and O-GlcNAc. In the LLC-shLKB1 group, compared with the LLC-shCtrl group, the expression levels of p53 and Caspase3 were reduced, while those of PCNA, BCL-2 and O-GlcNAc were elevated. Compared with the LLC-shLKB1 group, there was no statistically significant difference in p53, Caspase 3 and O-GlcNAc expression in the LLC-shLKB1+BIBR1532 group, while p53 and Caspase3 expression levels were increased in the LLC-shLKB1+DON group. While PCNA and BCL-2 expression levels were reduced in both the LLC-shLKB1+BIBR1532 group and the LLC-shLKB1+DON group. And O-GlcNAc expression levels were reduced in both the LLC-shLKB1+DON group and the combination therapy group. Additionally, the highest expression levels of p53 and Caspase3 were observed after combination therapy, while PCNA and BCL-2 expression was the lowest (Figure 7E). The heart, liver, spleen, lung and kidney from each group were stained with hematoxylin and eosin (H&E) to assess the effects on the organs of the mice. Neither monotherapy nor combination therapy caused organ damage in the mice (Supplementary Figure S5B). Moreover, both drugs effectively inhibited tumor growth in mice, with the combination treatment resulting in the most significant increase in antitumor activity.

**FIGURE 7:**
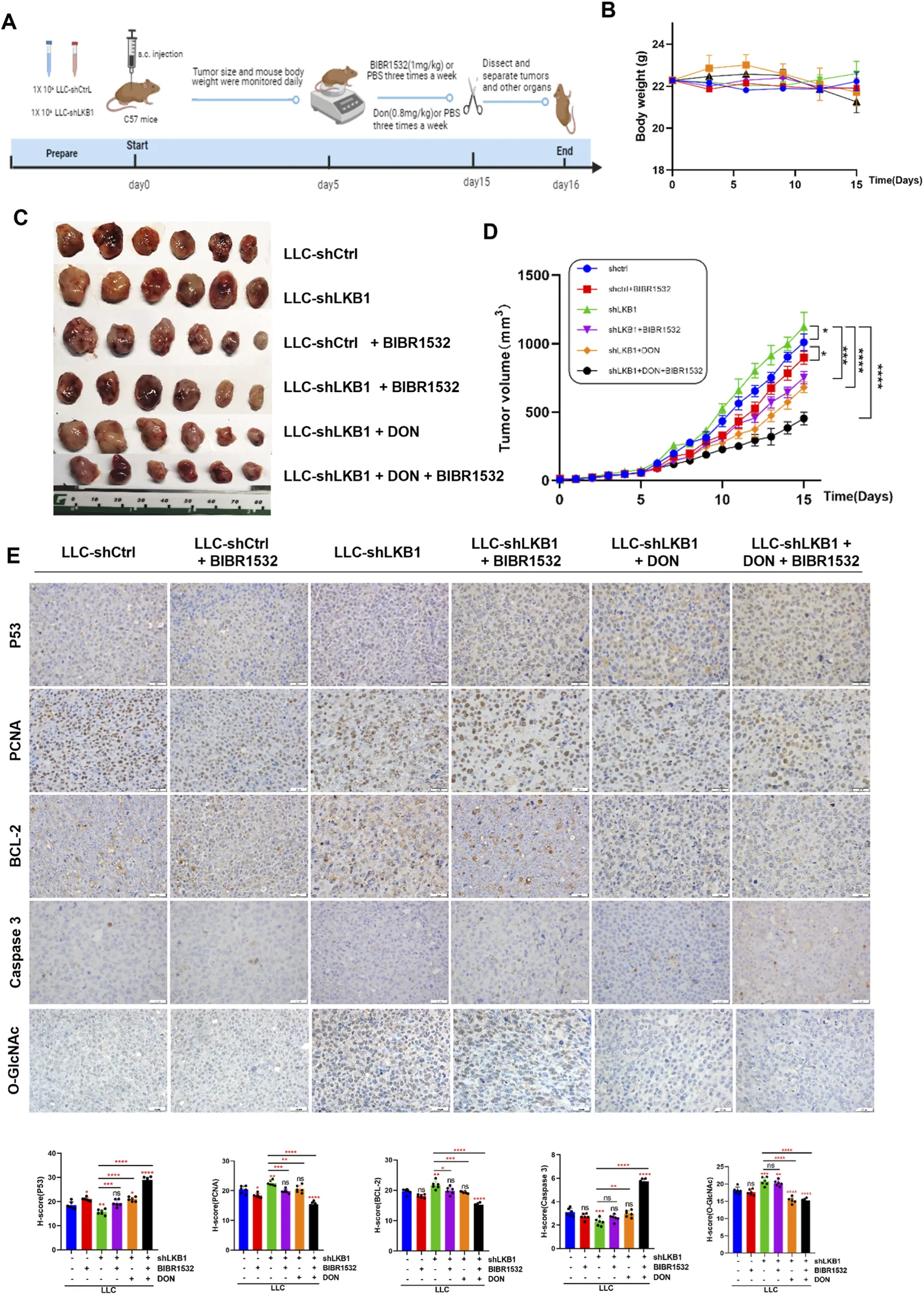
Evaluation of O-GlcNAc glycosylation-targeted drug efficacy in lung adenocarcinoma tumors. **(A)** Scheme of lung adenocarcinoma tumor transplantation and drug administration protocol. **(B)** Gross pictures of transplanted tumors in C57BL/6 mice. **(C)** Body weight growth curves of C57BL/6 mice. **(D)** Growth curves according to tumor volume. **(E)** Immunohistochemical staining to detect the expression of p53, BCL-2, and PCNA in transplanted tumor tissues (scale bar, 20 μm) and graphs of the results of the statistical analysis (normalized to Control). (n = 6, ns, *P* > 0.1, no significant difference; *, *P* < 0.05; **, *P* < 0.01; ***, *P* < 0.001; ****, *P* < 0.0001).

## 4 Discussion

This study provides a detailed elucidation of a novel signalling axis in lung adenocarcinoma, wherein LKB1 may downregulates O-GlcNAc glycosylation levels by phosphorylating GFAT1 *via* AMPK, thereby inhibiting telomerase activity and inducing telomere dysfunction and cellular senescence, along with its therapeutic implications. This mechanism closely links multiple biological processes, including energy metabolism, post-translational protein modification, chromosomal stability and cell fate determination. Malignant tumors primarily obtain energy through aerobic glycolysis *via* glucose, thereby sustaining tumor cells (Kesel, 2024). Elevated glucose uptake in LUAD cells increases the intracellular pool of glucose-6-phosphate, the primary substrate for the HBP branch pathway, which directly fuels the synthesis of UDP-GlcNAc, the nucleotide sugar donor for O-GlcNAc modification (Lunt and Vander Heiden, 2011). As another rate-limiting substrate for HBP, glutamine not only provides the amido group required for UDP-GlcNAc biosynthesis, but also supports tricarboxylic acid cycle anaplerosis and maintains intracellular NADPH homeostasis to counteract oxidative stress in rapidly proliferating LUAD cells (Hensley et al., 2013). Under glutamine deprivation, the elevated cellular AMP/ADP ratio robustly activates LKB1-AMPK signaling, leading to enhanced GFAT1 phosphorylation and functional inhibition, which restricts excessive HBP flux to prevent unnecessary consumption of limited metabolic resources. Under glucose starvation, the canonical energy-sensing cascade and the glycolytic metabolite-mediated LKB1 regulatory mechanism synergistically activate AMPK, rapidly suppress GFAT1 activity and downregulate global O-GlcNAc modification levels, redirecting limited metabolic substrates to core energy production processes (Hard and ie, 2014). Notably, LKB1-deficient LUAD cells lose this nutrient-sensing checkpoint, resulting in constitutive GFAT1 hyperactivation and aberrant O-GlcNAc accumulation even under nutrient limitation, which further drives malignant progression and therapeutic resistance (Murray et al., 2019). Telomere shortening, cellular senescence, oxidative stress, and oncogenic stress occur in precancerous tissues and during tumorigenesis and tumor progression, leading to molecular lesions. Sustained arrest of the cell cycle is induced in these lesions, which then prompts an immune clearance response. This ultimately prevents the malignant expansion of cancer cells (Ma et al., 2020) (Subedi et al., 2025). Somatic *LKB1* gene mutations are predominant in several cancer types, including lung cancer, and this gene plays a critical role as a tumor suppressor and metabolic regulator (Kim et al., 2017). Beyond its core cell-autonomous tumor-suppressive function, LKB1 inactivation represents one of the most clinically impactful co-mutational events in KRAS-driven lung adenocarcinoma. Patients harboring concurrent KRAS oncogenic activation and biallelic LKB1 loss define a distinct, highly aggressive LUAD subtype that accounts for approximately 25%–30% of all KRAS-mutant cases, and is consistently associated with accelerated tumor progression, substantially shortened progression-free survival, and markedly reduced objective response rates to PD-1/PD-L1 immune checkpoint monotherapy or chemo-immunotherapy combinations (Skoulidis et al., 2018) (Marabelle et al., 2020). This poor clinical outcome is closely tied to the unique metabolic rewiring imposed by LKB1 deficiency. LKB1 exerts its effects by phosphorylating AMPK and other substrates (Hollstein et al., 2019). AMPK, which consists of α, β and γ catalytic subunits, is widely recognized as the ‘master switch’ of metabolism (Freire-Agulleiro et al., 2025). Notably, a heightened intracellular ATP:ADP ratio is instrumental for cellular survival. A decrease in this ratio precipitates an immediate increase in AMP levels, indicating that the energy status of the cell has been compromised. In response, mechanisms that promote survival are activated, with AMPK serving as the central coordinator. Upon AMPK activation, energy consumption is restricted, and energy production is increased by the phosphorylation of various downstream targets to ensure cellular survival (Yanagi et al., 2024). Mammalian target protein of rapamycin (mTOR) is commonly targeted downstream of LKB1–AMPK to inhibit its function (Anand et al., 2020). The LKB1–AMPK axis has also been shown to effectively suppress tumorigenesis, and it determines metabolic plasticity and tolerance (Li et al., 2018). KRAS/LKB1 co-mutant tumors are characterized by profound metabolic inflexibility: they lose the canonical AMPK-dependent nutrient-sensing checkpoint, exhibit constitutively elevated glucose and glutamine uptake, and become critically dependent on non-canonical hexosamine biosynthesis pathway flux to sustain aberrant O-GlcNAcylation, telomerase hyperactivation, and uncontrolled senescence evasion under nutrient stress conditions (Shackelford and Shaw, 2009) (Kottakis et al., 2016). Importantly, this subtype also displays a distinctive immune-cold microenvironment, with marked reduction of infiltrating CD4^+^ and CD8^+^ effector T cells, accumulation of immunosuppressive neutrophils, and epigenetic silencing of the STING innate immune sensing pathway, further limiting the efficacy of currently approved first-line therapeutic regimens (Skoulidis et al., 2018). Recently, Natsume et al. extracted flavonoids from plants and demonstrated their ability to mediate AMPK phosphorylation, which in turn led to a notable increase in plasma membrane GLUT1 protein levels and *GLUT1* mRNA expression. This leads to increased glucose metabolism in skeletal muscle and other beneficial effects (Natsume et al., 2021). A further investigation revealed that the E6/E7 proteins of HPV16 markedly increase GLUT1 protein expression in lung carcinoma cells *via* a signaling cascade involving HPV, the LKB1 tumor suppressor protein (LKB1–HIF-1α–GLUT1) (Gu et al., 2019). Moreover, our study demonstrated that the induction of LKB1 expression, mediated by the addition of Tet to A549/Tet-LKB1 cells, resulted in an increase in GLUT1 protein levels. In this context, our current findings suggest that pharmacological suppression of O-GlcNAc signaling may represent a previously underappreciated strategy to selectively disrupt the acquired metabolic dependency of KRAS/LKB1 co-mutant LUAD. By blunting the unrestrained O-GlcNAc-mediated telomerase activation that these tumors rely on to bypass cellular senescence, this approach could preferentially impair the long-term proliferative capacity of LKB1-deficient tumor cells, while exerting comparatively milder effects on LKB1-proficient LUAD cells that retain intact metabolic checkpoint control. This subtype-selective vulnerability provides a strong mechanistic rationale for stratifying future preclinical and clinical studies of O-GlcNAc pathway inhibitors by LKB1 mutational status, to identify the patient subgroup most likely to derive maximal therapeutic benefit.

Protein O-GlcNAc glycosylation is defined as a reversible process that occurs posttranslationally and represents a response to external nutritional conditions and stress. The overall level of O-GlcNAc glycosylation increases in response to various cellular stressors, including drug therapies. However, the effects of chemotherapeutic agents on O-GlcNAc glycosylation in cancer cells are not yet fully understood, and further research will undoubtedly reveal the mechanisms involved. The process of O-GlcNAc glycosylation within cells is determined by a number of factors. The expression and activity of OGT and OGA play significant roles in this process (Cyriac and Lee, 2024). This study provides novel evidence that the inhibition of OGT and OGA enzyme activity modulates overall O-GlcNAc levels in tumor cells, with effects on telomere length and telomerase activity. The results also indicated that the inhibition of O-GlcNAc expression promoted tumor cell senescence. However, there was little change in the expression of the apoptotic protein BAX with the addition of OSMI-1 and thiamet-G. Moreover, the induction of LKB1 expression followed by the addition of thiamet-G did not result in any statistically significant difference in the expression of antiapoptotic protein BCL-2 compared with that after the induction of LKB1 expression mediated by the addition of tetracycline alone. These results indicate that the addition of these two drugs does not affect apoptosis in lung adenocarcinoma cells. Lee et al. reported that the coadministration of metformin and OSMI-1 to HCT116 colon cancer cells significantly upregulated BAX expression and downregulated BCL-2 levels, whereas the use of OSMI-1 alone had no effect on apoptosis (Lee et al., 2023). Moreover, Lee et al. demonstrated that combination therapy with doxorubicin and OSMI-1 significantly enhances apoptosis in hepatocellular carcinoma cells (HepG2). In contrast, OSMI-1 alone was not found to exert the same effects (Lee and Kwon, 2020). These findings strongly suggest that OSMI-1 and thiamet-G could serve as potential sensitizers for chemotherapy. Fundamental differences among cell lines in terms of origin, differentiation status and metabolic pathway activity may influence the effects of OSMI-1 and Thiamet-G on O-GlcNAc. The lung adenocarcinoma cell lines selected for this study (H1975, H1299 and A549) all harbour specific driver gene mutations. These mutations result in significant reprogramming of cellular metabolic pathways, leading to fundamental differences in sensitivity to changes in the extracellular environment compared to normal cells or other tumor cell types. These factors may explain the variations in the IC_50_ values of each drug observed across the three different cell lines in this study, as determined by the CCK-8 assay (Supplementary Figure S2A-F). Differences in the cellular microenvironment may also influence the toxicological response to the drugs. High concentrations of OSMI-1 and Thiamet-G may induce non-specific cytotoxicity rather than merely inhibitory effects. When doses exceed a certain threshold, changes in biological parameters resulting from cell death or damage may mask the specific effects of OGT and OGA inhibition, thereby causing the dose-response curve to deviate from expectations. Consequently, the Western blot results in this experiment also indicate that drug concentration is not dose-dependent (Supplementary Figure S3A-F).

The GFAT constitutes the initial stage of the HBP, functioning as the regulatory enzyme that modulates glucose levels (Yang et al., 2025). Among the identified human GFAT isoforms, GFAT1 is the most commonly expressed (Nabeebaccus et al., 2021). Moreover, GFAT1 is present at elevated levels in a variety of cancerous cell types, and its overexpression can be used to predict progression and poor pathological outcomes in different cancer types (Shimizu and Tanaka, 2019). This study demonstrated that LKB1 has no effect on GFAT1 protein expression in lung adenocarcinoma cells. However, the phosphorylation of GFAT1 was demonstrated *via* Co-IP assays. LKB1–AMPK-mediated regulation of O-GlcNAc glycosylation may be facilitated by the phosphorylation of GFAT1. This reduces its enzymatic activity, which in turn inhibits overall O-GlcNAc glycosylation. Zibrova et al. demonstrated that vascular endothelial growth factor inhibits GFAT1 enzyme activity through the AMPK-mediated phosphorylation of GFAT1, thereby alleviating the inhibitory effect of the HBP on angiogenesis (Zibrova et al., 2017). These findings suggest that the direct phosphorylation of GFAT1, meditated by AMPK, might play a role in regulating HBP dependence. Further research is required to determine how AMPK influences GFAT1 activity. On the basis of a literature, the site at which AMPK phosphorylates GFAT1 is Ser243 (Zibrova et al., 2017) (Scott and Oakhill, 2017); however, there is currently no commercially available antibody targeting this site. In the future, we intend to further validate this by performing quantitative Co-IP experiments or by collaborating with companies to develop anti-phospho-serine-targeting site-specific antibodies specific for the corresponding sites in subsequent experiments.

Senescent cells exhibit a range of distinctive phenotypic traits, including altered cell morphology with increased flattening and enlargement, elevated levels of reactive oxygen species (ROS), increased activity of the SA-β-gal enzyme, metabolic reprogramming, and altered transcriptional levels of multiple cyclin-dependent kinase (CDK) inhibitors (Zhou et al., 2023). To meet the bioenergetic and biosynthetic needs of the cell, cell cycle progression is linked to active anabolism, which requires substantial amounts of glucose and glutamine (Cyriac and Lee, 2024). The inhibition of telomerase activity is a promising new strategy for treating tumors, as tumor cells can proliferate indefinitely due to the activation of telomerase activity. Accordingly, numerous experiments have demonstrated that the inhibition of telomerase activity can induce apoptosis and inhibit the growth of lung cancer cells (Piñeiro-Hermida et al., 2023). Cancer senescence can be induced by oncogenes or treatments. Although this process can prevent cancer progression by inhibiting the proliferation of targeted cancer cells, the metabolism of senescent cells remains active. Research has indicated that the acute administration of MST-312 and BIBR1532 has the potential to reduce TERT expression, consequently resulting in telomere dysfunction and the activation of the DDR pathway (Fernandes et al., 2023). However, there are currently no reports on the combined application of DON and BIBR1532 for cancer treatment. In this study, we used a combination of these two drugs for the first time to treat C57BL/6 mice. The results showed that the combined application was more effective for lung adenocarcinoma treatment than either agent alone. These findings provide a foundation for the clinical treatment of lung adenocarcinoma. Nevertheless, further investigation is needed to ascertain whether this combination strategy exerts a similar tumor-suppressive effect on other tumor types.

This study has several limitations. First, the functional validation of the O-GlcNAc regulatory axis relied primarily on pharmacological modulation using OSMI-1, Thiamet-G and DON, while no corresponding genetic manipulation of OGT or OGA was performed. Consequently, potential off-target effects resulting from pharmacological intervention cannot be entirely ruled out. Future studies involving OGT/OGA knockdown or overexpression experiments are required to further confirm the specificity of this regulatory axis. Second, this study is limited by the lack of *in vitro* kinase assay and mass spectrometry evidence to formally confirm the direct, site-specific phosphorylation of GFAT1 by AMPK, which will be addressed in subsequent independent investigations. Third, the current study focuses on the molecular mechanism of LKB1 regulating O-GlcNAc-telomerase axis, and the systematic drug sensitivity comparison between LKB1-deficient and proficient cells will be the core content of our subsequent independent translational research. Fourth, identifying specific O-GlcNAc modification sites in the TERT protein and investigating their impact on protein stability and nuclear translocation will form the core focus of our future in-depth research. Fifth, we have not yet fully elucidated the exact causal relationship between LKB1-induced cellular senescence and the concurrently observed apoptosis phenotype. While senescence serves as the core mechanistic endpoint of this work, and apoptosis was only included as an auxiliary supplementary readout to profile the overall cellular response, the current experimental design cannot definitively distinguish whether the detected apoptosis acts as a secondary, delayed consequence triggered by accumulated senescence, or represents an independent parallel signaling pathway directly activated by LKB1 downstream cascades. Follow-up time-course kinetic analysis and senescence-specific genetic blockade assays will be required to resolve this regulatory hierarchy, which will further refine the biological interpretation of LKB1-mediated tumor suppression in lung adenocarcinoma. Furthermore, the detection of senescence markers (such as p16INK4a, Lamin B1, persistent DNA damage foci and SASP factors) is relatively limited. Future studies will incorporate more markers to conduct a more comprehensive analysis. Sixth, the suboptimal specificity and potential normal tissue toxicity of existing O-GlcNAc pathway inhibitors, substantial tumor heterogeneity across patient subgroups, and the fact that all current findings are validated only in cell line and conventional xenograft models. Further verification in patient-derived models and clinical sample cohorts will be required to advance these mechanistic insights toward future clinical application. Moreover, future application of highly selective GFAT or OGT inhibitors, with minimized off-target effects and improved *in vivo* safety, will provide a far more precise and actionable therapeutic approach for LKB1-deficient lung adenocarcinoma. Finally, large-scale clinical cohort analyses using TCGA and other public LUAD genomic resources will be conducted in follow-up studies, to clarify the clinical correlations between LKB1 status, O-GlcNAc pathway components, telomerase-related genes and patient prognosis, and further extend the translational implications of this work.

In summary, this study is the first to systematically reveal the pivotal role of the novel ‘LKB1-AMPK-GFAT1-O-GlcNAc-telomerase’ signalling axis in regulating senescence in lung adenocarcinoma cells, and organically integrates basal metabolism, protein modification and classical telomeric biology. This study not only deepens our understanding of the mechanisms underlying metabolic dependence and senescence escape in lung cancer, but more importantly, proposes targeting ‘O-GlcNAc modification’ in tumor cells as a novel therapeutic paradigm to overcome chemoresistance and induce programmed senescence in tumor cells. Future research should focus on elucidating the specific molecular details of this pathway, optimising combination drug regimens, and actively promoting translational medical research and early-phase clinical trials targeting LKB1-mutant lung cancer and other tumors with high O-GlcNAc levels.

## Data Availability

The raw data supporting the conclusions of this article will be made available by the authors, without undue reservation.
