# Supplementary material for: LKB1 promotes lung adenocarcinoma cell senescence by suppressing the O-GlcNAc glycosylation-mediated activation of telomerase
Source: Front Pharmacol. 2026 Sep 28;17:1952846. doi: 10.3389/fphar.2026.1952846 (PMC13655064; doi:10.3389/fphar.2026.1952846)
Supplement: Supplementary file 1 [file Supplementaryfile1.pdf]

Supplementary Materials

LKB1 promotes lung adenocarcinoma cell senescence by suppressing the O-GlcNAc glycosylation-mediated activation of telomerase

Liting Gu[1], Mingdi Liu[2], Yunkuo Li[2], Honglan Zhou[2,*], Yishu Wang[1,*], and Zhi-Xiang Xu[1,*]

1 Key Laboratory of Pathobiology, Ministry of Education, Jilin University, Changchun 130021, Jilin, China

2 Department of Urology, the First Hospital of Jilin University, Changchun 130021, Jilin, China

* **Correspondence**: Honglan Zhou, hlzhou@jlu.edu.cn; Yishu Wang, wangys@jlu.edu.cn; Zhi-Xiang Xu, zhixiangxu@jlu.edu.cn

Supplementary Figure 1

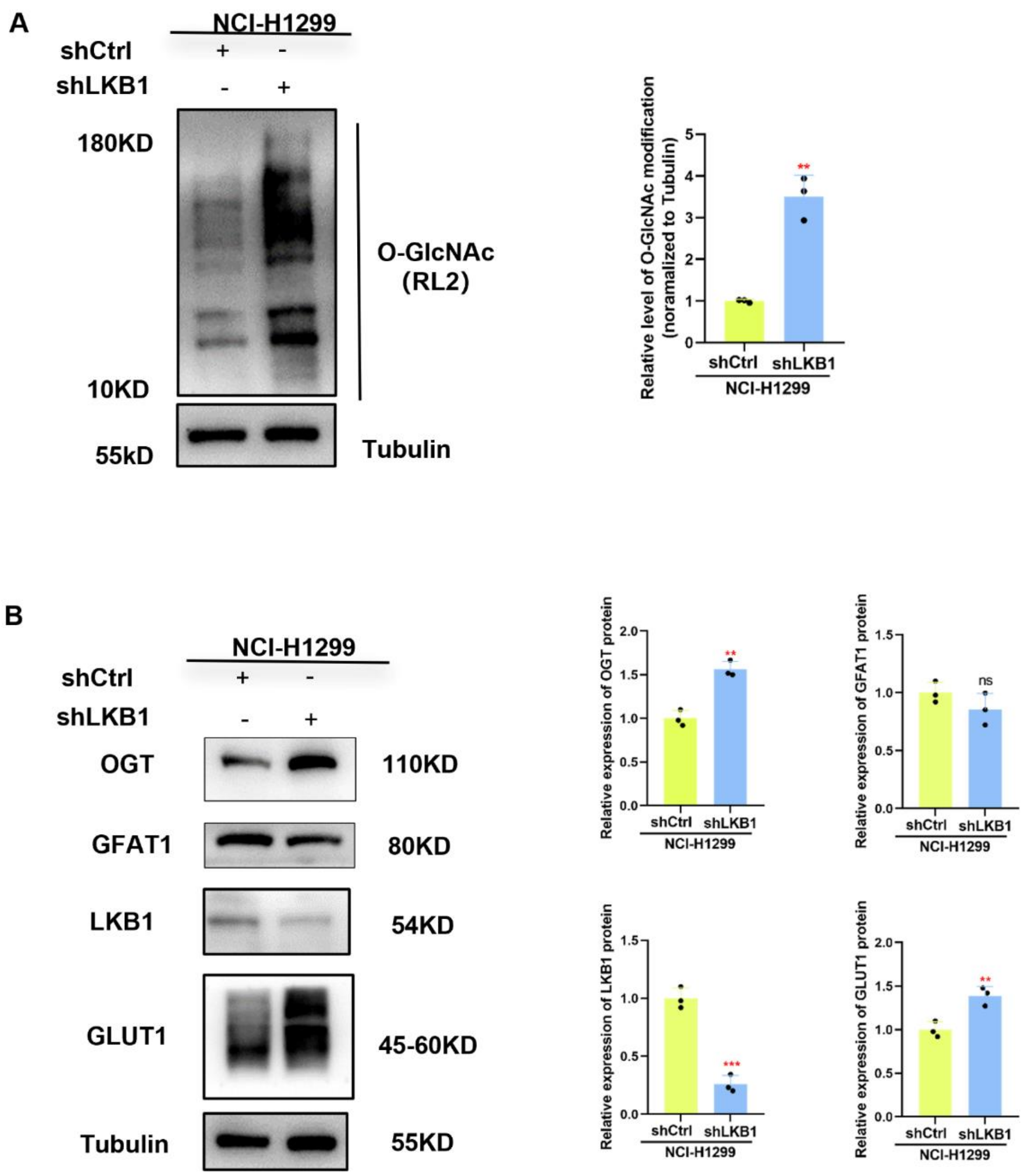

Supplementary Fig. 1 LKB1 inhibits O-GlcNAc glycosylation in NCI-H1299 cells

A. Western blot to detect the expression level of RL2 protein in NCI-H1299 cells and statistical graph of **(A); B.** Western blot to detect the expression level of OGT, GFAT1, LKB1, GLUT1, GAPDH protein in NCI-H1299 cells and statistical graph of **(B)** (normalized to Tubulin) (n=3, ns, $P > 0.1$, no statistical difference, *, $P < 0.05$, **, $P < 0.01$, ***, $P < 0.001$, ****, $P < 0.0001$).

Supplementary Figure 2

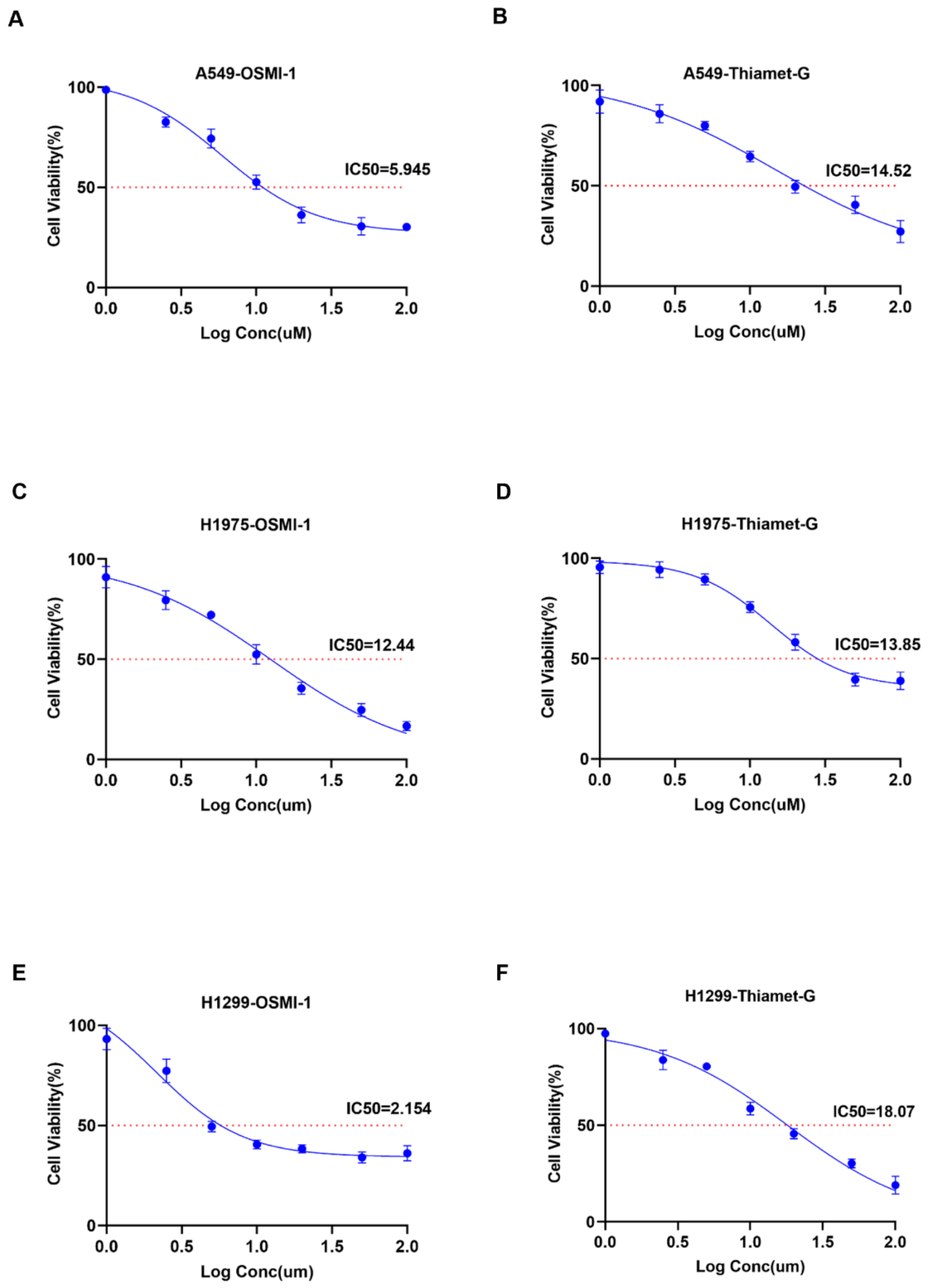

Supplementary Fig. 2 IC50 values of OSMI-1 and Thiamet-G in LUAD cells detected by CCK8 assay

A-B. IC50 values of OSMI-1**(A)** and Thiamet-G**(B)** in A549/Tet-LKB1 cells**; C-D.** IC50 values of OSMI-1 **(C)** and Thiamet-G in NCI-H1975 cells **(D); E-F.** IC50 values of OSMI-1 **(E)** and Thiamet-G **(F)** in NCI-H1299 cells.

Supplementary Figure 3

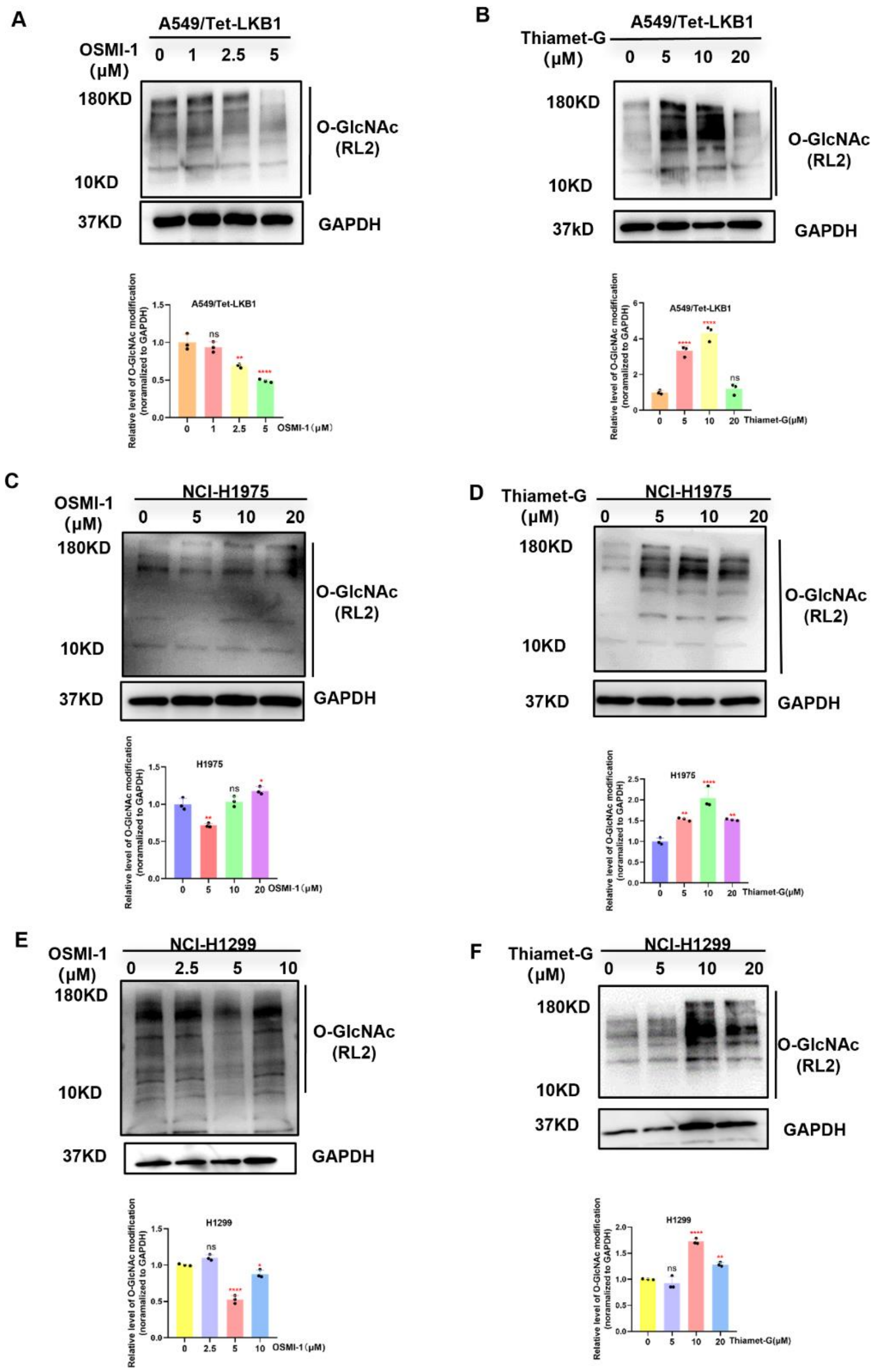

Supplementary Fig. 3 Effect of different concentrations of OSMI-1 and Thiamet-G on overall O-GlcNAc.

A-B. Western blot to detect the effect of different concentrations of OSMI-1**(A)** and Thiamet-G**(B)**on O-GlcNAc in A549/Tet-LKB1 cells and statistical plots of **(A,B)**; **C-D.** Western blot to detect the effect of different concentrations of OSMI-1**(C)** and Thiamet-G **(D)** on O-GlcNAc in NCI-H1975 cells and statistical plots of **(C, D)**; **E-F.** Western blot to detect the effect of different concentrations of OSMI-1**(E)** and Thiamet-G **(F)** on O-GlcNAc in NCI-H1299 cells and statistical plots of **(E, F)** (normalized to GAPDH) (n=3, ns, $P > 0.1$, no statistical difference, *, $P < 0.05$,**, $P < 0.01$, ****, $P < 0.0001$).

Supplementary Figure 4

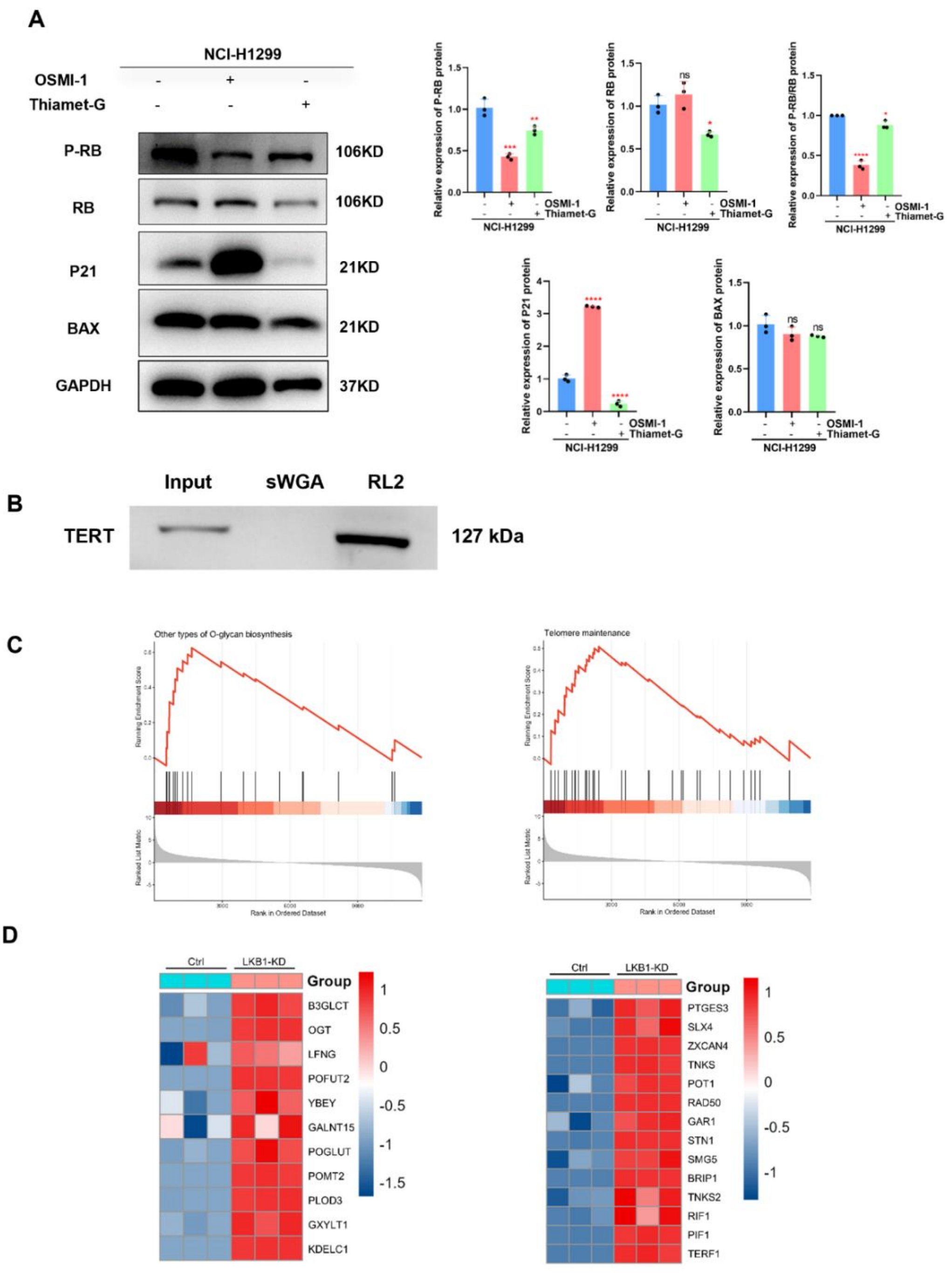

Supplementary Fig. 4 O-GlcNAc glycosylation inhibits cellular senescence in NCI-H1299 cells, and LKB1 regulates O-GlcNAc biosynthesis and telomere maintenance.

A. Western blot to detect p21, RB, P-RB, BAX, and GAPDH protein expression in NCI-H1299 cells and statistical plots (normalized to GAPDH); **B.** IP experiment to detect O-GlcNAc modification of TERT in A549 cells. **C-D.** Analysis of GSEA and heatmaps employing GSE51266 datasets has revealed that LKB1 regulates the O-GlcNAc biosynthesis and telomere maintenance pathways. (n=3, ns, $P > 0.1$, no statistical difference, *, $P < 0.05$, **, $P < 0.01$, ****, $P < 0.0001$).

Supplementary Figure 5

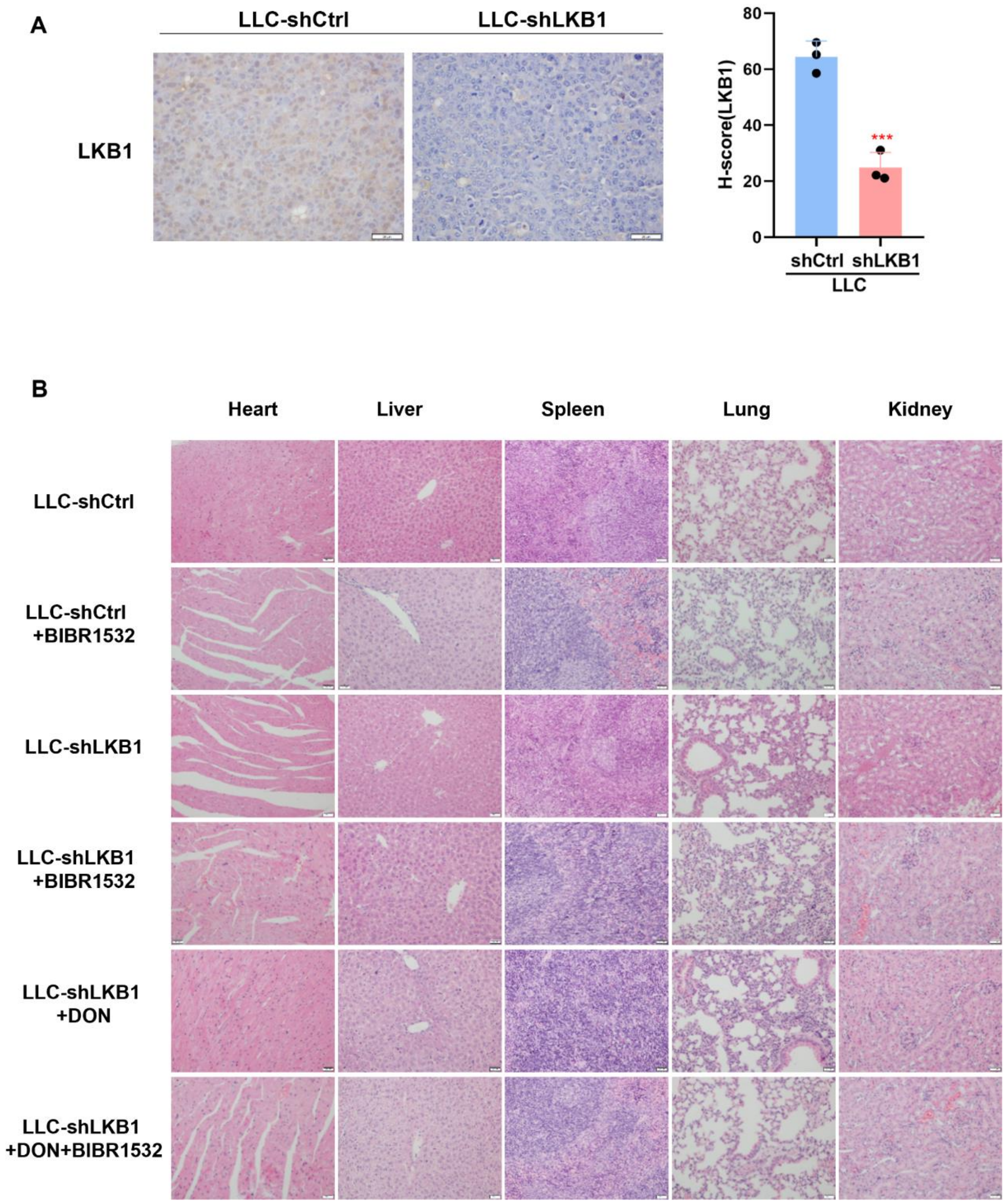

Supplementary Fig.5 IHC of Tumors in Mouse Tissue and HE Staining of Mouse Organs

A.IHC staining to detect the expression of LKB1 in grafted tumor tissues (Scale bar, 20 μm) and statistical graph (n=3, ***, $P < 0.001$). **B.** HE staining of organs of transplanted tumor mice to evaluate O-GlcNAc glycosylation targeted drug efficacy in lung adenocarcinoma tumors (Scale bar, 20 μm)

Supplementary Table1. The drug concentrations in this experiment for different cells utilized in this study

Cell types	Drug	concentration	treatment timing
A549/Tet-LKB1	OSMI-1	5 μM	24h
A549/Tet-LKB1	thiamet-G	10 μM	24h
NCI-H1975	OSMI-1	5 μM	24h
NCI-H1975	thiamet-G	10 μM	24h
NCI-H1299	OSMI-1	5 μM	24h
NCI-H1299	thiamet-G	10 μM	24h
A549/Tet-LKB1	tetracycline	1 μM	24h
A549/Tet-LKB1	CompoundC	5μM	24h
A549/Tet-LKB1	A-769662	0.8μM	24h

Supplementary Table2. Sources and catalogue numbers of the drugs used in this study

Drug	Source	Catalog number
OSMI-1	MedChemExpress	HY-119738
Thiamet-G	MedChemExpress	HY-119738
tetracycline	Apexbio	C6589
Compound C	MedChemExpress	HY-13418A
A769662	MedChemExpress	HY-50662
DON	MedChemExpress	HY-108357
BIBR1532	MedChemExpress	HY-17353

Supplementary Table3. Primers utilized in this study

Gene	Forward primer	Reverse primer
H-GAPDH	CAAGGTCATCCATGACAACTTTG	GTCCACCACCCTGTTGCTGTAG
H- STK11	GCACCCTCAAAATCTCCGAC	CAAGCATCCCTTTCAGCAGG
H-OGA	TGTTTCTGGGCCCGTACAAA	CTCCACTGTGTGCAACTTGC
H-OGT	CGGCTGCGTGTAGGATATGT	TCCTGATGGATGCGATCAGC
H-TUBA1B	TGCTCATGGAACGTCTCTCA	TCAGGTCAACATTCAGGGCT
H-GFPT1	AGTCCTGTCAATAGCCACCC	GCCCGGGAAAATGAACACTT
H-IL-1B	TTCGACACATGGGATAACGA	TCTTTCAACACGCAGGACAG
H-CCL5	CGCTGTCATCCTCATTGCTA	GAGCACTTGCCACTGGTGTA
H- SERPINE1	ATGGGATTCAAGATTGATGA	CAGGGGTGGTTATTGCATCT

Supplementary Table 4 Antibodies utilized in this study.

Antibody	Application	Source	Catalog number
LKB1	WB	Cell Signaling Technology	3047S
LKB1	IHC	Cell Signaling Technology	13031S
RL2	WB	Invitrogen	MA1-072
GFAT1	WB	Cell Signaling Technology	3818
GLUT1	WB	Cell Signaling Technology	73015
OGT	WB	Abcam	ab96718
RB	WB	ABclonal Technology	A28031
P-RB	WB	ABclonal Technology	AP1419
Phosphotyrosine	WB	PTM BIO	PTM701
OGA	WB	Abcam	ab124807
GAPDH	WB	Proteintech	60004-1-Ig
Tubulin	WB	Proteintech	66240-1-Ig
PCNA	IHC	Cell Signaling Technology	13110
cleaved-Caspase 3	IHC	Cell Signaling Technology	9579
BCL2	WB	Proteintech	60178-1-lg
BAX	WB	Proteintech	60267-1-lg
P53	WB/IHC	Proteintech	60283-2-lg
BCL2	IHC	Proteintech	68103-1-lg
AMPK	WB	Cell Signaling Technology	5831
P-AMPK	WB	Cell Signaling Technology	2535
P21	WB	Cell Signaling Technology	2947S
TERT	WB	Abcam	ab32020

Supplementary table5. A summary of the individual data points, exact p-values and detailed statistical methods for all statistical figures in this study.

Figure number	Protein name	statistical test	Control group data (n=3/6)	Treatment Group 1 (n=3/6)	Treatment Group 2 (n=3/6/6)	Treatment Group 3 (n=3/6)	Treatment Group 4 (n=3/6)	Treatment Group5 (n=3/6)	p-value
**Fig.1B**	O-GlcNAc	Student's t test	0.994222<br>1.000165<br>1.005614	0.550456<br>0.618528<br>0.532455					<0.0001
	OGA	Student's t test	1.121423<br>0.9925<br>0.966077	0.716418<br>1.016835<br>0.883505					0.1948
	OGT	Student's t test	1.0151708<br>0.9848292<br>1.0000000	0.997369<br>1.096118<br>1.116743					0.1379
	GFAT1	Student's t test	1.0151708<br>0.9848292<br>1.0000000	1.121745<br>0.921626<br>1.039229					0.6636
	LKB1	Student's t test	1.0151708<br>0.9848292<br>1.0000000	1.5993<br>1.714521<br>1.638523					<0.0001
	GLUT1	Student's t test	1.0151708<br>0.9848292<br>1.0000000	3.102496<br>3.209106<br>3.158638					<0.0001
**Fig.1D**	O-GlcNAc	Student's t test	1.02313544<br>1.03183988<br>0.94502468	1.390342<br>1.547052<br>1.42628					0.0012
	OGA	Student's t test	0.906667<br>0.953312<br>1.040021	1.228654<br>1.098173<br>0.846189					0.4865
	OGT	Student's t test	0.858938<br>1.203858<br>0.957205	0.743417<br>1.061806<br>1.137849					0.8793
	GFAT1	Student's t test	0.858938<br>1.203858<br>0.957205	1.211815<br>0.879589<br>1.078778					0.7403
	LKB1	Student's t test	0.858938<br>1.203858<br>0.957205	0.381397<br>0.530485<br>0.449206					0.0077
	GLUT1	Student's t test	0.858938<br>1.203858<br>0.957205	7.076729<br>8.613959<br>7.189556					0.0002

**Fig.1E**	OGA	Student's t test	1.069091<br>0.905659<br>1.02525	0.942227<br>0.803982<br>0.853571					0.1032
	OGT	Student's t test	0.976515<br>1.007365<br>1.01612	0.945143<br>0.917481<br>0.978638					0.0685
	GFAT1	Student's t test	0.976515<br>1.007365<br>1.01612	0.988612<br>0.942115<br>0.842389					0.1665
	LKB1	Student's t test	0.976515<br>1.007365<br>1.01612	1.455343<br>1.351625<br>1.413667					0.0002
	GLUT1	Student's t test	0.976515<br>1.007365<br>1.01612	1.241171<br>1.257227<br>1.328524					0.0007
**Fig.1F**	OGA	Student's t test	0.938255<br>1<br>1.061745	1.289357<br>1.108611<br>1.127864					0.0602
	OGT	Student's t test	0.970209<br>1.032526<br>0.997265	0.87439<br>0.934319<br>0.912867					0.0210
	GFAT1	Student's t test	0.970209<br>1.032526<br>0.997265	0.910404<br>1.031273<br>1.047669					0.9433
	LKB1	Student's t test	0.970209<br>1.032526<br>0.997265	0.643277<br>0.684594<br>0.661215					<0.0001
	GLUT1	Student's t test	0.970209<br>1.032526<br>0.997265	1.144407<br>1.364766<br>1.31328					0.0165
**Fig2A**	A549-β-gal	One way ANOVA	1.012024<br>0.993988<br>0.993988	1.157302<br>1.313294<br>1.213294	0.626725<br>0.560183<br>0.578583				0.0034<br>0.0001
**Fig2B**	H1975-β-gal	One way ANOVA	1.035362<br>1.064944<br>0.899694	1.586087<br>1.656579<br>1.427478	0.662632<br>0.621217<br>0.882782				0.0021<br>0.0477
**Fig.2C**	P-RB	One way ANOVA	1.016449<br>1.038466<br>0.945084	0.5975516<br>07<br>0.5957505<br>35<br>0.4553042<br>39	1.33936<br>1.220737<br>1.423989				0.0012<br>0.0060
	RB	One way ANOVA	0.868926<br>1.133412	1.5875613<br>45	0.781782<br>0.665792				0.0004<br>0.0253

			0.997663	1.7441948<br>63<br>1.7441948<br>63	0.646629				
	P-<br>RB/RB	One way<br>ANOVA	1.15167491<br>3<br>0.91622993<br>2<br>0.94729783<br>5	0.476396<br>0.441562<br>0.36104	1.313214<br>1.233511<br>1.202173				<0.0001<br>0.0012
	P53	One way<br>ANOVA	1.104754<br>0.986913<br>0.908333	1.4405763<br>9<br>1.3386484<br>03<br>1.2625160<br>53	0.618411<br>0.442041<br>0.571512				0.0092<br>0.0024
	P21	One way<br>ANOVA	0.977517<br>0.907752<br>1.114731	1.1629844<br>99<br>1.2474614<br>01<br>1.2618069<br>14	0.568517<br>0.462559<br>0.605541				0.0331<br>0.0011
	BAX	One way<br>ANOVA	0.988375<br>1.084918<br>0.926707	1.0158200<br>92<br>0.9008996<br>72<br>0.8235362<br>84	0.871165<br>1.090929<br>0.827237				0.6190<br>0.7231
**Fig.2D**	IL-1β	One way<br>ANOVA	0.951285<br>0.933883<br>1.114831	1.478133<br>1.73379<br>1.684332	0.53776<br>0.61832<br>0.616548				0.0006<br>0.0060
	CCL5	One way<br>ANOVA	0.951285<br>0.933883<br>1.114831	1.428173<br>1.215021<br>1.33232	0.860361<br>0.760639<br>0.921754				0.0110<br>0.1699
	SERPIN<br>E1	One way<br>ANOVA	0.951285<br>0.933883<br>1.114831	1.534148<br>1.638363<br>1.495443	0.816804<br>0.769274<br>0.676466				0.0003<br>0.0192
**Fig.2E**	IL-1β	One way<br>ANOVA	1.183471901<br>0.850382512<br>1.026145587	1.529375<br>1.365745<br>1.302116	0.649402<br>0.6853<br>0.701198				0.0140<br>0.0220
	CCL5	One way<br>ANOVA	1.183471901<br>0.850382512<br>1.026145587	1.74178<br>1.862118<br>1.551949	0.546731<br>0.629387<br>0.612043				0.0013<br>0.0148
	SERPIN	One way	1.18347190	1.870601	0.438619				0.0013

	E1	ANOVA	1<br>0.850382512<br>1.026145587	1.60421<br>1.936992	0.339504<br>0.537735				0.0059
**Fig.2F**	A549-EdU	One way ANOVA	1.01178<br>1.032134<br>0.956086	0.625344<br>0.694611<br>0.68089	1.165592<br>1.190471<br>1.242374				0.0001<br>0.0017
	H1975-EdU	One way ANOVA	0.9<br>1<br>1.1	0.86931<br>0.813532<br>0.726809	1.18551<br>1.180108<br>1.245479				0.0306<br>0.0266
**Fig.3A**	TRAP	One way ANOVA	1.034914507<br>0.964829681<br>1.002923648	0.514207014<br>0.508161782<br>0.562880212	1.174933<br>1.206911<br>1.146499				<0.0001<br>0.0009
**Fig.3C**	TRF	One way ANOVA	0.892355<br>1.052226<br>1.055419	0.389266<br>0.242875<br>0.371483	1.59906<br>1.383149<br>1.420189				0.0003<br>0.0019
**Fig.3E**	Q-Fish	One way ANOVA	1.1110047<br>0.9370137<br>0.9519816	1.607307<br>1.714498<br>1.5912	0.764803<br>0.741626<br>0.815789				<0.0001<br>0.0145
**Fig.3F**	Q-Fish	One way ANOVA	1.1110047<br>0.9370137<br>0.9519816	1.700082<br>1.72507<br>1.799599	0.440746<br>0.671162<br>0.555954				0.0001<br>0.0018
**Fig.3G**	Q-Fish	One way ANOVA	1.1110047<br>0.9370137<br>0.9519816	1.788631<br>1.613227<br>1.754033	0.808704<br>0.761471<br>0.667015				0.0001<br>0.0214
**Fig.4A**	A549-O-GlcNAc	One way ANOVA	1.089191<br>1.037283<br>0.970755	0.632536<br>0.716818<br>0.707509	2.598282<br>2.241193<br>2.681801	0.399065<br>0.440074<br>0.447328			0.0360<br><0.0001<br>0.0015<br><0.0001
	H1975-O-GlcNAc	One way ANOVA	1.068859<br>0.979146<br>0.991995	1.41746<br>1.50503<br>1.48209	1.4094351<br>1.3975865<br>1.3191188	0.755028<br>0.743325<br>0.554288			0.0002<br>0.0010<br>0.0019<br><0.0001
**Fig.4B**	A549-β-gal	One way ANOVA	1.000466<br>1.029736<br>0.969798	1.241085<br>1.296595<br>1.288856	0.839678<br>0.82372<br>0.855247	1.206021<br>1.196344<br>1.174045			<0.0001<br>0.0002<br><0.0001<br><0.0001
	H1975-β-gal	One way ANOVA	1.091918<br>0.906379<br>1.001702	0.681612<br>0.707519<br>0.660355	0.724565<br>0.655879<br>0.681156	0.99465<br>0.835589<br>0.962828			0.0017<br>0.0018<br>0.5995

									0.0077
**Fig.4C**	P-RB	One way ANOVA	1.123591<br>0.926351<br>0.950058	0.649761<br>0.722988<br>0.779526	1.112002<br>1.076149<br>1.022606	0.577883<br>0.457179<br>0.500962			0.0066<br>0.6598<br>0.0002<br>0.0017
	RB	One way ANOVA	0.991828<br>1.006802<br>1.00137	1.392785<br>1.544119<br>1.447228	0.878566<br>0.729887<br>0.955891	1.718284<br>1.619924<br>1.866346			0.0013<br>0.2945<br><0.0001<br>0.0002
	P-RB/RB	One way ANOVA	1.132348639<br>0.920092531<br>0.948758201	0.766519<br>0.86822<br>0.738634	1.265701<br>1.274405<br>1.169794	0.436314<br>0.382222<br>0.368419			0.0032<br>0.0014<br><0.0001<br><0.0001
	P53	One way ANOVA	0.952574<br>0.947926<br>1.109501	1.20477<br>1.141846<br>1.1888	0.801466<br>0.848997<br>0.721877	1.332688<br>1.346543<br>1.313709			0.0273<br>0.0097<br>0.0006<br>0.0002
	P21	One way ANOVA	0.952574<br>0.947926<br>1.109501	1.319892<br>1.27951<br>1.374277	1.076132<br>0.859009<br>1.054568	1.394356<br>1.372583<br>1.403724			0.0049<br>0.9996<br>0.0015<br>0.0043
	BCL-2	One way ANOVA	0.907612<br>1.104043<br>1.040944	0.856302<br>0.866247<br>0.884642	0.864765<br>0.820199<br>0.804686	1.004686<br>1.078976<br>1.01311			0.0500<br>0.0156<br>0.9881<br>0.8319
	LKB1	One way ANOVA	1.00000<br>1.00000<br>1.00000	1.192785<br>1.144119<br>1.147228	1.208284<br>1.119924<br>1.216346	0.956302<br>0.966247<br>0.984642			0.0009<br>0.0004<br>0.6282<br>0.8530
**Fig.4D**	P-RB	One way ANOVA	0.981124<br>0.932347<br>1.186529	1.88589<br>2.327213<br>2.343225	3.022147<br>3.229397<br>3.413229	1.59477<br>1.421668<br>1.642166			0.0003<br><0.0001<br>0.0361<br>0.0131
	RB	One way ANOVA	1.033398<br>1.095753<br>0.870849	0.629412<br>0.42874<br>0.551528	0.908793<br>0.879002<br>0.879411	1.315999<br>1.212233<br>1.261336			0.0005<br>0.4001<br>0.0179<br><0.0001
	P-RB/RB	One way ANOVA	0.949415<br>1.050873<br>1.162497	3.996273<br>4.428029<br>4.248606	3.325451<br>3.673936<br>3.881267	1.011832<br>1.172768<br>0.901926			<0.0001<br><0.0001<br>0.9985<br><0.0001
	P53	One way	1.020129	0.866546	0.581346	0.828058			0.0121

		ANOVA	1.047161<br>0.93271	0.826206<br>0.822829	0.470651<br>0.531778	0.871803<br>0.903912			<0.0001<br>0.0344<br>0.8648
	P21	One way<br>ANOVA	0.93642<br>1.158692<br>0.924888	0.473569<br>0.538568<br>0.499065	0.647384<br>0.556615<br>0.501835	0.879522<br>1.143367<br>0.943405			0.0015<br>0.0037<br>0.9964<br>0.0019
	BAX	One way<br>ANOVA	1.076924<br>0.940065<br>0.983011	0.609295<br>0.81445<br>0.66871	0.998461<br>0.891829<br>0.793419	0.555589<br>0.490044<br>0.454406			0.0107<br>0.4741<br>0.0004<br>0.0844
	LKB1	One way<br>ANOVA	1.000000<br>1.000000<br>1.000000	0.969412<br>0.89874<br>0.851528	1.208793<br>1.169002<br>1.159411	0.8915999<br>0.882233<br>0.861336			0.0362<br>0.0008<br>0.0090<br>0.7351
**Fig.4E**	A549-<br>IL-1β	One way<br>ANOVA	1.062289<br>1.041367<br>0.896344	1.518986<br>1.394112<br>1.230902	0.661307<br>0.69382<br>0.695495	1.352743<br>1.627765<br>1.444526			0.0124<br>0.0329<br>0.0034<br>0.0003
	A549-<br>CCL5	One way<br>ANOVA	1.062289<br>1.041367<br>0.896344	1.550428<br>1.465867<br>1.695304	1.186695<br>1.259928<br>1.274293	1.338934<br>1.450136<br>1.30839			0.0002<br>0.0362<br>0.0035<br>0.0066
	A549-<br>SERPIN<br>E1	One way<br>ANOVA	1.062289<br>1.041367<br>0.896344	1.60465<br>1.50558<br>1.480212	0.716288<br>0.801148<br>0.75031	1.38127<br>1.205064<br>1.346329			0.0001<br>0.0180<br>0.0045<br><0.0001
**Fig.4F**	A549-<br>EdU	One way<br>ANOVA	1.077484<br>0.944389<br>0.978127	0.72419<br>0.660868<br>0.546356	0.877587<br>1.002483<br>0.990552	0.538659<br>0.43213<br>0.479742			0.0013<br>0.8794<br><0.0001<br>0.0030
	H1975-<br>EdU	One way<br>ANOVA	0.993232<br>1.036543<br>0.970224	1.613804<br>1.416315<br>1.347964	1.416315<br>1.373004<br>1.53785	1.223941<br>1.124759<br>1.295237			0.0014<br>0.0018<br>0.0861<br>0.0493
**Fig.5A**	A549-<br>TRAP	One way<br>ANOVA	0.9<br>1<br>1.1	0.8044035<br>03<br>0.7971720<br>7<br>0.8184244<br>27	1.028116<br>1.018387<br>1.040927	0.80986<br>0.822459<br>0.805528			0.0070<br>0.8933<br>0.0084<br>0.0030
**Fig.5B**	H1975-<br>TRAP	One way<br>ANOVA	1.085719<br>0.914281<br>1	1.559439<br>1.409178<br>1.674309	1.442642<br>1.521679<br>1.482161	0.99898<br>0.725432<br>0.862206			0.0010<br>0.0024<br>0.4368

									0.0002
**Fig.5D**	TRF	One way<br>ANOVA	1.018036<br>0.972993<br>1.008971	0.842495<br>0.777832<br>0.797821	1.018218<br>1.110399<br>1.025067	0.717701<br>0.643119<br>0.651439			0.0012<br>0.4185<br><0.0001<br>0.0002
**Fig.5E**	Q-Fish	One way<br>ANOVA	0.98461539<br>1<br>1.01538461	2.707693<br>2.811834<br>2.759763	1.068826<br>1.014633<br>1.10044	2.436923<br>2.38914<br>2.358313			<0.0001<br>0.3089<br><0.0001<br><0.0001
	Q-Fish	One way<br>ANOVA	0.98461539<br>1<br>1.01538461	1.662234<br>1.41012<br>1.520686	0.795804<br>1.038712<br>0.917258	1.778369<br>1.823729<br>1.86909			0.0004<br>0.6909<br><0.0001<br>0.0002
	Q-Fish	One way<br>ANOVA	0.98461539<br>1<br>1.01538461	1.678934<br>1.410489<br>1.544711	0.955493<br>0.779481<br>0.867486	1.64557<br>1.673942<br>1.702313			0.0002<br>0.2700<br><0.0001<br><0.0001
**Fig.6B**	O-<br>GlcNAc	One way<br>ANOVA	1.007055<br>0.992945<br>1	0.722105<br>0.692818<br>0.701686	0.928009<br>0.99486<br>0.961435	0.857609<br>0.842794<br>0.899442	0.901419<br>0.89486<br>0.90814		<0.0001<br>0.2537<br>0.0001<br>0.0016<br><0.0001
**Fig.6C**	LKB1	One way<br>ANOVA	1.1318424<br>1.0664329<br>0.8017247	1.5749141<br>3<br>1.6211089<br>7<br>1.5185205<br>2	1.86325983<br>9<br>1.83348058<br>8<br>1.81451605	1.024569<br>0.894527<br>0.878762	0.722091<br>0.719251<br>0.874149		0.0002<br><0.0001<br>0.9113<br>0.0986<br>0.0480
	P-<br>AMPK	One way<br>ANOVA	1.1318424<br>1.0664329<br>0.8017247	1.635912<br>1.46617<br>1.74576	1.304021<br>1.177033<br>1.307775	2.276285<br>2.485474<br>2.357287	1.540688<br>1.59233<br>1.534073		0.0005<br>0.1131<br><0.0001<br>0.0012<br>0.0263
	AMPK	One way<br>ANOVA	1.1318424<br>1.0664329<br>0.8017247	1.445453<br>0.996315<br>1.220883	1.116903<br>0.714886<br>0.981544	1.414163<br>1.252776<br>1.070872	1.12065<br>0.753586<br>0.937118		0.6397<br>0.9940<br>0.5501<br>0.9938<br>0.4250
	P-<br>AMPK/<br>AMPK	One way<br>ANOVA	1<br>1<br>1	1.531764<br>1.471593<br>1.429916	1.167533<br>1.246463<br>1.332365	2.109634<br>1.983973<br>2.201278	1.874816<br>2.013004<br>1.937012		<0.0001<br>0.0120<br><0.0001<br><0.0001<br>0.0201
	GFAT1	One way	1.1318424	0.9952109	0.94836600	0.929054	0.905829		0.6836

		ANOVA	1.0664329<br>0.8017247	0.8563588<br>8<br>0.7628313<br>2	3<br>0.95445267<br>7<br>1.02986530<br>2	0.831685<br>0.788171	0.86612<br>0.648829		0.9992<br>0.5569<br>0.3361<br>0.8063
**Fig.6D**	TRAP	One way<br>ANOVA	1<br>1<br>1	0.7952109<br>0.7563588<br>8<br>0.7628313<br>2	0.94836600<br>3<br>0.95445267<br>7<br>1.02986530<br>2	0.629054<br>0.631685<br>0.688171	1.1318424<br>1.2664329<br>1.3017247		0.0013<br>0.9767<br><0.0001<br>0.0011<br>0.0027
**Fig.7E**	P53	One way<br>ANOVA	17.77<br>17.064<br>17.278<br>18.1995<br>19.586<br>20.962	20.44<br>20.85<br>21.083<br>20.196<br>22.241<br>21.02	15.053<br>17.02<br>14.086<br>16.086<br>16.5995<br>15.103	18.762<br>17.437<br>19.0995<br>18.796<br>20.988<br>21.041	21.188<br>22.003<br>20.796<br>19.996<br>20.003<br>21.976	27.677<br>29.482<br>28.241<br>30.041<br>30.295<br>27.982	0.0095<br>0.0027<br>0.7768<br>0.0087<br><0.0001<br><0.0001<br><0.0001<br><0.0001
	PCNA	One way<br>ANOVA	21.636<br>20.651<br>20.364<br>19.273<br>18.658<br>21.544	18.6388<br>17.957<br>18.418<br>19.6388<br>17.623<br>18.1309	23.9312<br>21.99<br>22.0896<br>23.364<br>21.651<br>22.364	19.673<br>19.658<br>19.584<br>20.14<br>18.957<br>21.057	19.14<br>20.34<br>19.662<br>21.606<br>20.151<br>22.364	15.6388<br>14.623<br>15.1309<br>16.662<br>14.957<br>15.957	0.0125<br>0.0036<br>0.9317<br>0.9992<br><0.0001<br>0.0003<br>0.0091<br><0.0001
	BCL-2	One way<br>ANOVA	19.94<br>20.34<br>19.862<br>20.158<br>18.084<br>19.4188	18.6388<br>17.957<br>18.418<br>17.636<br>18.651<br>17.364	23.9312<br>21.99<br>22.0896<br>20.036<br>21.051<br>21.264	21.636<br>20.651<br>20.364<br>19.673<br>19.158<br>18.0584	19.673<br>19.658<br>19.584<br>19.0388<br>19.157<br>18.918	15.6388<br>14.623<br>15.1309<br>16.04<br>15.34<br>14.862	0.0544<br>0.0033<br>0.9922<br>0.9914<br><0.0001<br>0.0145<br>0.0007<br><0.0001
	Caspase<br>3	One way<br>ANOVA	3.576<br>2.745<br>2.945<br>2.823<br>3.156<br>3.384	2.703<br>2.684<br>2.6935<br>2.957<br>2.99<br>2.277	2.432<br>1.97<br>2.751<br>2.323<br>2.245<br>1.876	2.957<br>2.623<br>2.556<br>2.703<br>2.123<br>2.732	3.213<br>3.054<br>3.324<br>2.7388<br>2.93115<br>2.583	5.766<br>5.957<br>5.973<br>5.6388<br>5.351<br>5.62	0.2027<br>0.0002<br>0.0575<br>0.9659<br><0.0001<br>0.3003<br>0.0020<br><0.0001
	O-	One way	17.94	16.6388	21.9312	21.636	15.673	15.6388	0.9036

	GlcNAc	ANOVA	18.34<br>19.862<br>17.158<br>18.084<br>17.4188	16.957<br>18.418<br>17.636<br>18.651<br>17.364	20.99<br>19.0896<br>20.036<br>22.051<br>20.264	20.651<br>20.364<br>19.673<br>19.158<br>20.0584	16.658<br>15.584<br>14.0388<br>15.157<br>14.918	14.623<br>15.1309<br>16.04<br>15.34<br>14.862	0.0002<br>0.0029<br><0.0001<br><0.0001<br>0.9363<br><0.0001<br><0.0001
**SF1A**	O-GlcNAc	Student's t test	0.956245<br>1.023478<br>1.020277	2.937396<br>3.63585<br>3.940527					0.0011
**SF1B**	OGT	Student's t test	0.979167<br>0.920482<br>1.100351	1.500083<br>1.666823<br>1.517908					0.0017
	GFAT1	Student's t test	0.979167<br>0.920482<br>1.100351	0.996932<br>0.721948<br>0.852509					0.2088
	LKB1	Student's t test	0.979167<br>0.920482<br>1.100351	0.203329<br>0.344671<br>0.231839					0.0004
	GLUT1	Student's t test	0.979167<br>0.920482<br>1.100351	1.420991<br>1.272908<br>1.479269					0.0085
**SF3A**	O-GlcNAc	One way ANOVA	0.914755<br>0.965993<br>1.119253	0.872134<br>0.933735<br>1.01001	0.6615562<br>0.7097835<br>0.697133	0.48269<br>0.510863<br>0.468941			0.5530<br>0.0011<br><0.0001
**SF3B**	O-GlcNAc	One way ANOVA	0.914755<br>0.965993<br>1.119253	3.498797<br>3.453166<br>2.968253	4.508706<br>4.605637<br>3.831451	1.322293<br>1.409687<br>0.844331			<0.0001<br><0.0001<br>0.7828
**SF3C**	O-GlcNAc	One way ANOVA	0.975066<br>1.090883<br>0.934051	0.746459<br>0.702526<br>0.696048	1.036287<br>0.968331<br>1.10712	1.239174<br>1.165576<br>1.139625			0.0011<br>0.7985<br>0.0164
**SF3D**	O-GlcNAc	One way ANOVA	0.975066<br>1.090883<br>0.934051	1.564205<br>1.491016<br>1.5467	2.337474<br>1.885746<br>1.904693	1.500437<br>1.545674<br>1.518292			0.0034<br><0.0001<br>0.0040
**SF3E**	O-GlcNAc	One way ANOVA	1.017018<br>0.991563<br>1	1.14595<br>1.091867<br>1.068908	0.582113<br>0.472582<br>0.527347	0.840419<br>0.938196<br>0.853072			0.0563<br><0.0001<br>0.0191
**SF3F**	O-GlcNAc	One way ANOVA	1.008437<br>0.991563<br>1	1.067802<br>0.85301<br>0.85301	1.697743<br>1.785704<br>1.689419	1.328611<br>1.275377<br>1.252795			0.4581<br><0.0001<br>0.0028
**SF4A**	P-RB	One way ANOVA	1.131864<br>0.924943<br>0.993193	0.393016<br>0.463203<br>0.42818	0.695682<br>0.797434<br>0.744092				0.0001<br>0.0060
	RB	One way	1.131864	1.169096	0.661636				0.3616

		ANOVA	0.924943<br>0.993193	1.270161<br>0.969431	0.644243<br>0.705784				0.0143
	P-RB/RB	One way ANOVA	1.000000<br>1.000000<br>1.000000	0.336171<br>0.364681<br>0.441682	0.851457<br>0.937785<br>0.854277				<0.0001<br>0.0276
	P21	One way ANOVA	1.131864<br>0.924943<br>0.993193	3.190631<br>3.245078<br>3.217909	0.158231<br>0.323844<br>0.237024				<0.0001<br><0.0001
	BAX	One way ANOVA	1.131864<br>0.924943<br>0.993193	0.88363<br>0.993193<br>0.833371	0.865564<br>0.891473<br>0.877891				0.2044<br>0.1208
**SF5A**	IHC-LKB1	Student’s t test	69.571<br>58.537<br>65.222	21.1<br>31.129<br>22.18					0.0009
